# CircRNome‐wide characterisation reveals the promoting role of circAATF in anti‐PD‐L1 immunotherapy of gallbladder carcinoma

**DOI:** 10.1002/ctm2.70060

**Published:** 2024-10-20

**Authors:** Yueqi Wang, Shengli Li, Xiaobo Bo, Yuan Li, Changcheng Wang, Lingxi Nan, Dexiang Zhang, Houbao Liu, Jiwei Zhang

**Affiliations:** ^1^ Department of Biliary Surgery Zhongshan Hospital Fudan University Shanghai China; ^2^ Biliary Tract Diseases Institute, Fudan University Shanghai China; ^3^ Cancer Center, Zhongshan Hospital, Fudan University Shanghai China; ^4^ Precision Research Center for Refractory Diseases and Shanghai Key Laboratory of Pancreatic Diseases, Shanghai General Hospital, Shanghai Jiao Tong University School of Medicine Shanghai China; ^5^ Shanghai Key Laboratory of Compound Chinese Medicines, The MOE Key Laboratory for Standardization of Chinese Medicines, Institute of Chinese Materia Medica, Shanghai University of Traditional Chinese Medicine Shanghai China; ^6^ Department of General Surgery Xuhui District Central Hospital of Shanghai Shanghai China

**Keywords:** circular RNA, gallbladder carcinoma, immunotherapy, PD‐L1, tumour immunology

## Abstract

Circular RNAs (circRNAs) have been shown to play important roles in tumour development and tumour immunology. However, genome‐wide characterisation of circRNAs and their roles in the immunology and immunotherapy of gallbladder carcinoma (GBC) has been lacking. We present a comprehensive characterisation of the circRNA landscape in GBC, revealing GBC‐specific circRNAs. Our analysis found that circRNAs are significantly enriched in cell proliferation and are involved in cancer‐related hallmarks. In particular, circAATF was upregulated in GBC, which was positively correlated with AATF mRNA expression, and promoted GBC cell growth. Through integrating computational and experimental approaches, we revealed that circAATF is positively associated with the CD4^+^ T cell abundance and PD‐L1 level, and enhances the clinical benefits of anti‐PD‐L1 immunotherapy for GBC. We further demonstrate that circAATF elevates the PD‐L1 level by activating phosphorylated AKT and acting as a sponge for miR‐142‐5p. CircAATF is positively associated with CD4^+^ T cells and PD‐L1 levels and shows potential to aid anti‐PD‐L1 immunotherapy for GBC. Our study provides insights into roles of circAATF in the tumour development and immunology of GBC and accelerates the development of therapeutic strategies for GBC immunotherapy.

**Highlights:**

- We present a comprehensive characterisation of circRNA landscape in gallbladder carcinoma (GBC).
- CircAATF is positively associated with CD4^+^ T cell abundance and PD‐L1 expression and is shown to promote PD‐L1 treatment in mouse model.
- CircAATF can elevate PD‐L1 level through phosphorylated AKT and linear AATF, which upregulates PD‐L1 by acting as a sponge of miR‐142‐5p.

## Graphical abstract

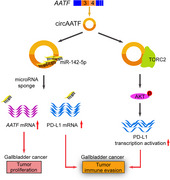

## 1 INTRODUCTION

Gallbladder carcinoma (GBC) is the most common biliary tract malignancy, an intensely aggressive neoplasm that accounts for more than 80% of biliary tract tumour.^1^, ^2^ The incidence of GBC shows a prominent geographical variation around the world, with higher rates found in developing regions, such as East Asian and South American countries. Currently, surgical resection is the only effective treatment for GBC patients, and adjunctive chemotherapy and radiotherapy show minimal prognostic improvement.^3^ Unfortunately, most GBC patients are diagnosed at advanced tumour stages and with early metastasis, leading to a poor prognosis and a median survival of less than one year.^4^ Despite the increasing application of immunotherapy in various cancers, its use in GBC remains limited.^5^, ^6^, ^7^ Over the past decade, monoclonal antibodies targeting programmed cell death protein 1 (PD‐1), such as pembrolizumab, nivolumab, and cemiplimab, as well as those targeting programmed cell death ligand 1 (PD‐L1), including durvalumab and atezolizumab, have been explored in a range of solid tumours.^5^, ^6^ However, reliable biomarkers to predict the efficacy of immunotherapy are still lacking.^6^ Therefore, more efforts are needed to elucidate the underlying molecular mechanisms to improve our understanding of GBC development and further facilitate the discovery of novel and effective diagnostic and therapeutic targets for patients with GBC.

The advent of deep RNA sequencing (RNA‐seq) technology has enabled researchers to characterise transcriptional events that contribute to the diversity of the transcriptome in human cancers, including alternative splicing,^8^ back‐splicing,^9^ and alternative polyadenylation.^10^ Among these transcriptional events, back‐splicing covalently links a downstream splice‐donor site to an upstream splice‐acceptor site to generate circular RNAs (circRNAs),^11^ which have been shown to play crucial roles in human cancers,^12^, ^13^, ^14^, ^15^ including GBC.^16^, ^17^ Wang et al. identified circROBO1 in paired samples from primary breast cancer and its liver metastasis, demonstrating that circROBO1 promotes carcinogenesis and liver metastasis of breast cancer via the circROBO1/KLF5/FUS feedback loop.^14^ Additionally, the circRNA circ0030018 was found to be downregulated by isoliquiritigenin, which inhibits glioma tumourigenesis and progression.^13^ Using RNA sequencing on four GBC tissues and paired normal adjacent tissues (NATs), Wang et al. identified circFOXP1, which was further demonstrated to be positively associated with lymph node metastasis and poor prognosis in GBC patients.^16^ They also revealed the biological role of circFOXP1 as a sponge for miR‐370 to regulate gene *PKLR* in GBC progression. In addition, circERBB2 was shown to be significantly upregulated in GBC tissues and to promote GBC cell proliferation.^17^ Genome‐wide characterisation of the circRNA transcriptome (CircRNome) has been shown to be effective in uncovering molecular mechanisms and discovering candidate targets for precision therapy in human diseases.^12^, ^18^, ^19^, ^20^ Despite examinations of individual circRNAs, few studies have fully characterised the CircRNome in GBC using high‐depth RNA sequencing technology.

Growing evidence has shown that tumour cells can acquire the capability to evade immune surveillance,^21^ which is one of the major mechanisms underlying the uncontrolled rapid growth of malignant tumours. Therefore, a promising therapeutic strategy is to activate the immune response against tumour cells, where immune checkpoint blockade (ICB) has proven to be the most promising approach to stimulate antitumour immunity.^22^ Furthermore, recent studies have shed lights on the roles of circRNAs in the immune response,^23^, ^24^, ^25^ suggesting that circRNAs might be significant factors in regulating immunity and potential immunotherapy targets in human cancers. However, little is known about how circRNAs regulate tumour immunity in GBC.

In this study, we present a comprehensive characterisation of CircRNome in GBC, which enhances in‐depth understanding of the transcriptional diversity of GBC. CircRNAs were found to be closely associated with cell proliferation and cancer signalling in GBC. We revealed that the majority of differentially expressed circRNAs were positively correlated with their parental genes in GBC, such as circAATF. CircAATF was significantly upregulated in GBC and promoted tumour development in GBC. We further found that circAATF was positively associated with CD4^+^ T cell abundance and PD‐L1 expression in GBC and was shown to aid PD‐L1 treatment in humanised GBC mouse models. Additionally, our analysis revealed that circAATF could elevate PD‐L1 levels through modulation of AKT phosphorylation and acting as a sponge for miR‐142‐5p, which targets both circAATF and PD‐L1 gene. Our exploration of the roles of circRNAs in GBC tumour immunology will facilitate the development of potential targets for immunotherapy for GBC patients.

## 2 MATERIALS AND METHODS

### 2.1 Sample collections and cell lines

Primary gallbladder carcinoma tissues and their corresponding normal adjacent tissues (NATs) were obtained from surgical specimens at Fudan University Zhongshan Hospital in Shanghai, China. This study involved 50 primary gallbladder carcinoma tissues with corresponding NATs, selected based on pathological diagnosis criteria, referred to as Cohort 1 (n = 50). From this cohort, 20 sample pairs were randomly chosen for RNA sequencing, and 15 pairs for quantitative PCR (qPCR). The Clinical Research Ethics Committee of Fudan University Zhongshan Hospital approved the study, and all tissues were obtained with written informed content from patients in accordance with institutional review board protocols.

The cell lines used in this study include GBC‐SD, SGC‐996, NOZ, OCUG, and G415, sourced from the Cell Bank of the Chinese Academy of Sciences (Shanghai, China). GBC cells were cultured in DMEM with 1% penicillin‐streptomycin and 10% fetal bovine serum, while HEK‐293T cells were maintained in RPMI 1640 with the same supplements. All experimental procedures were conducted in accordance with the Declaration of Helsinki and strictly adhered to the guidelines for animal care.

### 2.2 RNA sequencing

Total RNA samples (3 µg) were extracted using TRIzol reagent and subsequently processed with the RiboMinus Eukaryote Kit (Qiagen, Valencia, CA) to eliminate ribosomal RNA before RNA sequencing library preparation. The NEBNext Ultra Directional RNA Library Prep Kit for Illumina (NEB, Beverly, MA) was utilised as per the manufacturer's protocol. Approximately 50 ng of ribosome‐depleted RNA was fragmented, followed by complementary DNA (cDNA) synthesis using random hexamer primers for both strands. A dUTP mix facilitated the removal of the second strand. The cDNA was then subjected to end repair with the End‐It DNA End Repair Kit and modified with Klenow to append an adenine to the 3′ends prior to adaptor ligation.

Following ligation, the cDNA products were purified and treated with uracil DNA glycosylase to remove the second strand. The purified first‐strand cDNA underwent 13–16 cycles of PCR amplification. Library quality was assessed using a Bioanalyzer 2100 (Agilent, Santa Clara, CA), and sequencing was conducted on a HiSeq 2000 (Illumina, San Diego, CA, USA). Detailed statistics for each RNA‐seq library are available in Table S1. All raw sequencing data can be found in the Gene Expression Omnibus (GEO) database under accession number GSE138109.

### 2.3 Identification of circRNAs in GBC samples

To obtain a reliable circRNA repertoire in GBC, we employed four algorithms: find_circ,^26^ circRNA_finder,^27^ CIRI2,^28^ and CIRCexplorer2.^29^ The default parameters in each tool were adopted to detect circRNAs. For further analysis, we retained circRNAs that were detected by at least two methods and had a minimum of two back‐splicing reads. Additionally, circRNAs found in at least five samples from either tumour or NAT tissues were classified as high‐confidence.

For each of these high‐confidence circRNAs, we calculated the average number of back‐splicing reads across the methods as the raw expression level for the corresponding samples. This raw expression level was normalised based on the total reads in each sample. High‐confidence circRNAs were annotated with their host genes using coordinate intersection via BEDTools software.^30^

Furthermore, the raw counts of circRNAs in all samples were subjected to DESeq2^31^ to perform differential expression analysis. CircRNAs demonstrating a fold change of at least 1.5 and a Benjamini–Hochberg adjusted *p*‐value of less than .05 were regarded as significantly differentially expressed circRNAs in GBC.

### 2.4 Calculation of circRNA activity scores of biological hallmarks

All the back‐splicing reads within a gene were first summed and normalised by the total mapped reads in the corresponding sample. Then the normalised back‐spliced reads of genes in hallmarks were used to calculate a circRNA activity score. In particular, the GSVA software^32^ was used to derive an enrichment score for each hallmark gene set in each GBC sample. A positive GSVA score indicates an overall high back‐splicing activity for a certain hallmark in the corresponding sample. Additionally, the gene sets of hallmarks were retrieved from Molecular Signature Database (MSigDB).^33^

### 2.5 Flow cytometry

Cells were filtered through 40‐µm meshes to generate single‐cell suspensions for subsequent analysis. The cell surfaces were labelled using a panel of antibodies, including anti‐human CD4, Ki67, PD‐L1, pAKT(Ser473), and AATF (BioLegend, San Diego, CA). These antibodies were conjugated with fluorophores, including fluorescein isothiocyanate, PerCP/Cy5.5, phycoerythrin, and Brilliant Violet 421. Analysis was conducted using an Attune flow cytometer (Life Technologies), and the data were processed and interpreted with FlowJo software (Tree Star) to assess cell populations and their activation states.

### 2.6 Co‐culture of tumour cells with PBMCs

Human blood was collected, and PBMCs were isolated via Ficoll gradient centrifugation. Tumour cells in the logarithmic growth phase were harvested, counted, and adjusted to a density of 1 × 10^4^ cells per mL. PBMCs were similarly counted and adjusted to 1 × 10^5^ cells per well. The tumour cells and PBMCs were then co‐cultured for 48 h in a 96‐well plate prior to further experimentation.

### 2.7 In vivo assays

Female athymic NSG mice (5 to 6 weeks) were obtained from The Jackson Laboratory (Bar Harbor, ME, USA) and bred through sibling matings. PBMCs were isolated from healthy donors by collecting whole blood with preservative‐free heparin, diluting it (1:3) in low‐endotoxin PBS, and using Ficoll–Paque gradient centrifugation. After washing the PBMC layer with PBS, 3 × 10^6^ cells were injected into the tail vein of NSG mice to create a humanised model.

For subcutaneous tumour studies, groups of five mice received .2 mL of a suspension containing 1 × 10^6^ GBC‐SD cells from stable cell lines: pWPXL‐vector, pWPXL‐IgG, pWPXL‐AATF, and pWPXL‐circAATF, injected into the right axillary region. Tumour growth were monitored, and volume calculated using the formula: volume = (length × width^2^) × .5. For spleen in situ injection experiments, groups of five mice were given 50 µL of the same cell suspension. Tumour‐bearing mice received intraperitoneal injections of either isotype control IgG or anti‐PD‐L1 antibody (200 µg/mouse, clone 10F.9.G2, BioxCell) every three days, with no notable weight differences between the groups. All procedures adhered to the guidelines set by the Shanghai Medical Experimental Animal Care Commission.

### 2.8 Statistical analysis

Statistical analysis and data visualisation were conducted with GraphPad Prism 5 and R software (R Foundation for Statistical Computing, Vienna, Austria; http://www.r‐project.org). All tests were two‐tailed unless otherwise specified, with a *p*‐value of less than .05 indicating statistical significance.

## 3 RESULTS

### 3.1 Characterisation of the CircRNome in gallbladder carcinoma

**FIGURE 1:**
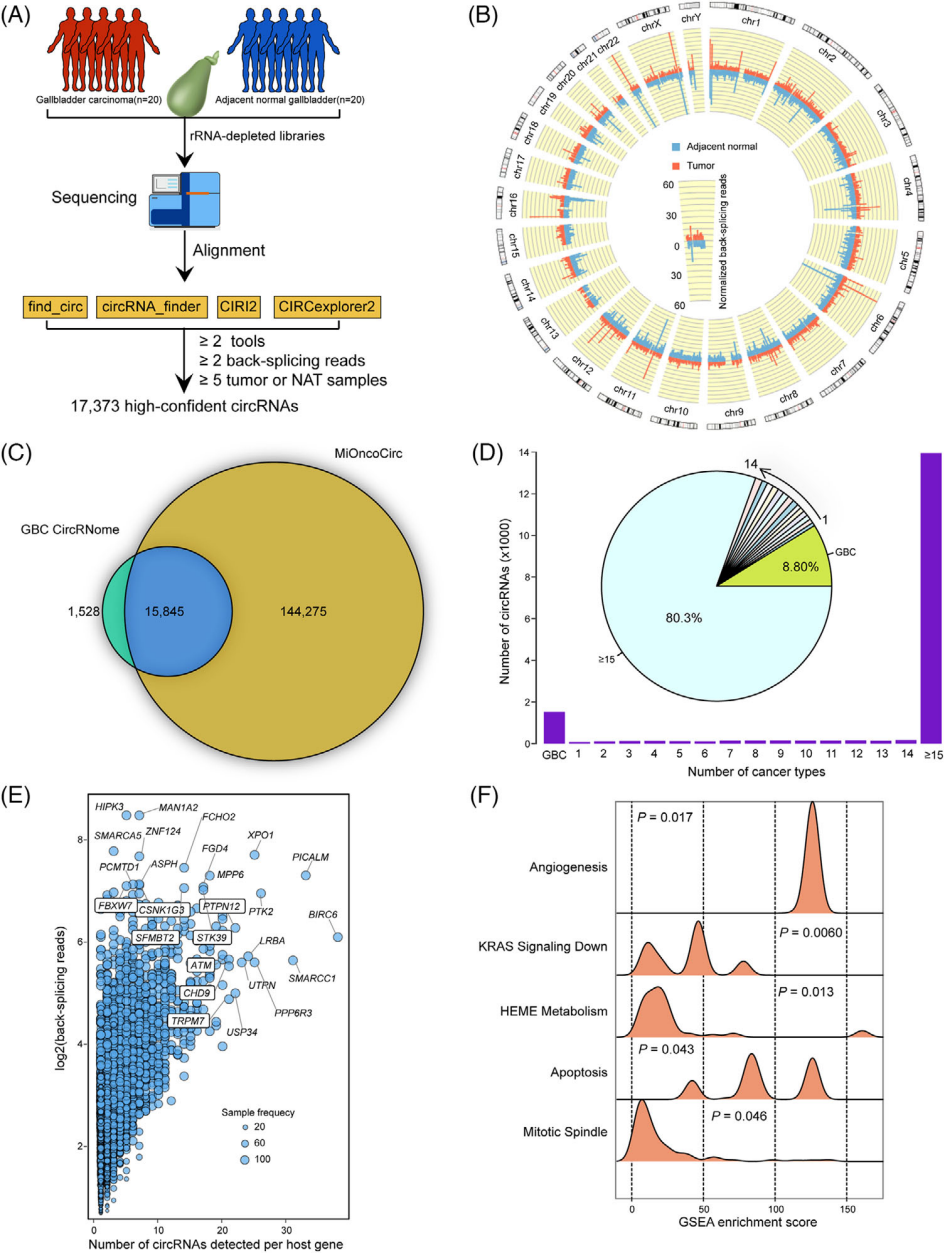
Characterisation of the CircRNome in GBC. (A) Schematic flowchart of circRNA identification in GBC. (B) Distribution of detected back‐splicing reads across the human chromosomes, with normalised back‐splicing reads calculated in 1Mb windows. (C) Overlap of identified circRNAs in GBC with high‐confidence circRNAs in the MiOncoCirc database. (D) Distribution of the number of cancer types where GBC circRNAs were detected. (E) Number of circRNAs and back‐splicing reads of corresponding genes. Circle size indicates the frequency of samples in which circRNAs were detected. (F) GSEA enrichment score density plot for genes generating circRNAs in GBC tumour samples.

To comprehensively characterise the landscape of circRNAs in GBC, we collected 20 GBC tumour tissues with matched NATs (Figure 1A). To identify high‐confidence circRNAs in GBC, four circRNA detection tools were employed to determine back‐splicing sites (see Section 2). On average, 416 and 372 normalised back‐splicing reads were detected in GBC tumour and NAT samples, respectively, showing no significant distribution bias (Figure S1A). The back‐splicing events occurred across all human chromosomes, with back‐splicing peaks appearing on chromosomes such as chromosome 22 and the chromosome X (Figure 1B). Moreover, staggered back‐splicing peaks appeared on chromosomes 14 and 16, indicating possible dysregulation of circRNAs in GBC. CircRNAs covered by at least 2 back‐splicing reads and in at least 5 different samples were considered robustly expressed. In total, 17 373 high‐confidence circRNAs were detected in GBC (Table S2), with the number of circRNAs ranging from 3121 to 10 187 across different samples (Figure S1B). To further examine the reliability of the detected circRNAs in GBC, we screened the circRNAs in the MiOncoCirc database, which characterises circRNAs across > 2000 cancer samples.^9^ A majority (91.2%, *n* = 15 845) of GBC circRNAs were present in the MiOncoCirc cohort (Figure 1C). Furthermore, most (80.3%) of the overlapped circRNAs were identified in more than 15 cancer types, suggesting that cancer‐ubiquitous circRNAs are widely expressed in GBC samples (Figure 1D). Notably, over 80% of GBC circRNAs were detected in the breast invasive carcinoma (BRCA), prostate adenocarcinoma (PRAD), and sarcoma (SARC) cohorts, while only a small portion were expressed in the skin cancer (SKIN), Langerhans cell histiocytosis (LCH) cohorts (Figure S1C). Approximately one‐third (30.4%) of the circRNAs identified in our data were also detected in the cholangiocarcinoma (CHOL) samples from the MiOncoCirc cohort (Figure S1D). Additionally, a considerable portion (8.80%) of the total circRNAs were identified as GBC‐specific, not detected in the other 44 cancer types. In summary, our screening provides a CircNome‐wide view of GBC.

We further examined the properties of circRNAs to enhance our understanding of back‐splicing events in GBC. Over 80% of the detected circRNAs were covered by 2–6 normalised back‐splicing reads, indicating their relatively low expression in GBC samples (Figure S2A). In total, 19 236 genes (32.9% of all annotated genes in GENCODE v28) were found to generate circRNAs (Figure S2B) in GBC, with the majority being protein‐coding genes (17 591 protein‐coding genes, 91.4% of all circRNA‐expressing genes). Genes exhibiting higher expression levels of back‐splicing were more likely to be detected in more GBC samples (Figure S2C). Specifically, the back‐splicing reads of genes detected in more than 10 samples were significantly higher than those in fewer than 10 samples (Figure S2D, *p* < 2.2E‐16, fold change = 2.5, Wilcoxon rank sum test). Genes *HIPK3* and *MAN1A2* exhibited the most abundant back‐splicing reads in GBC samples, while gene *BIRC6* was found to express the largest number of circRNAs (more than 30 circRNAs across GBC samples) (Figure 1E). To further explore the biological implications of circRNAs in GBC, we performed gene set enrichment analysis (GSEA) on genes that expressed circRNAs in GBC samples. These circRNA‐expressing genes were significantly enriched in tumour‐related biological hallmarks, such as ‘Mitotic spindle’, ‘Apoptosis’, ‘Heme metabolism’, ‘KRAS signalling’, and ‘Angiogenesis’ (Figure 1F). These results suggest that circRNAs may play important roles in the development of GBC. To investigate the specific biological hallmarks circRNAs may impact in GBC, circRNA activity scores were calculated for each hallmark in each sample (see Section 2). In addition to cell proliferation‐associated hallmarks, cancer signalling and immune‐related hallmarks were found to have high circRNA activity scores (Figure S2E). Specifically, 76.9% (10 out of 13) of cancer signalling hallmarks exhibited back‐splicing activity in at least half of the GBC samples, such as ‘KRAS signalling’ and ‘Hedgehog signalling’. Interestingly, 85.7% (6 out of 7) of immune‐associated hallmarks showed relatively high back‐splicing activity, such as ‘IL6/JAK/STAT3 signalling’ and ‘Inflammatory response’. In summary, our analysis reveals that cell proliferation, cancer signalling, and immune‐related hallmarks possess back‐splicing activity, indicating the potential roles of circRNAs in the tumour biology of GBC.

### 3.2 Transcriptional dysregulation of circRNAs in GBC

**FIGURE 2:**
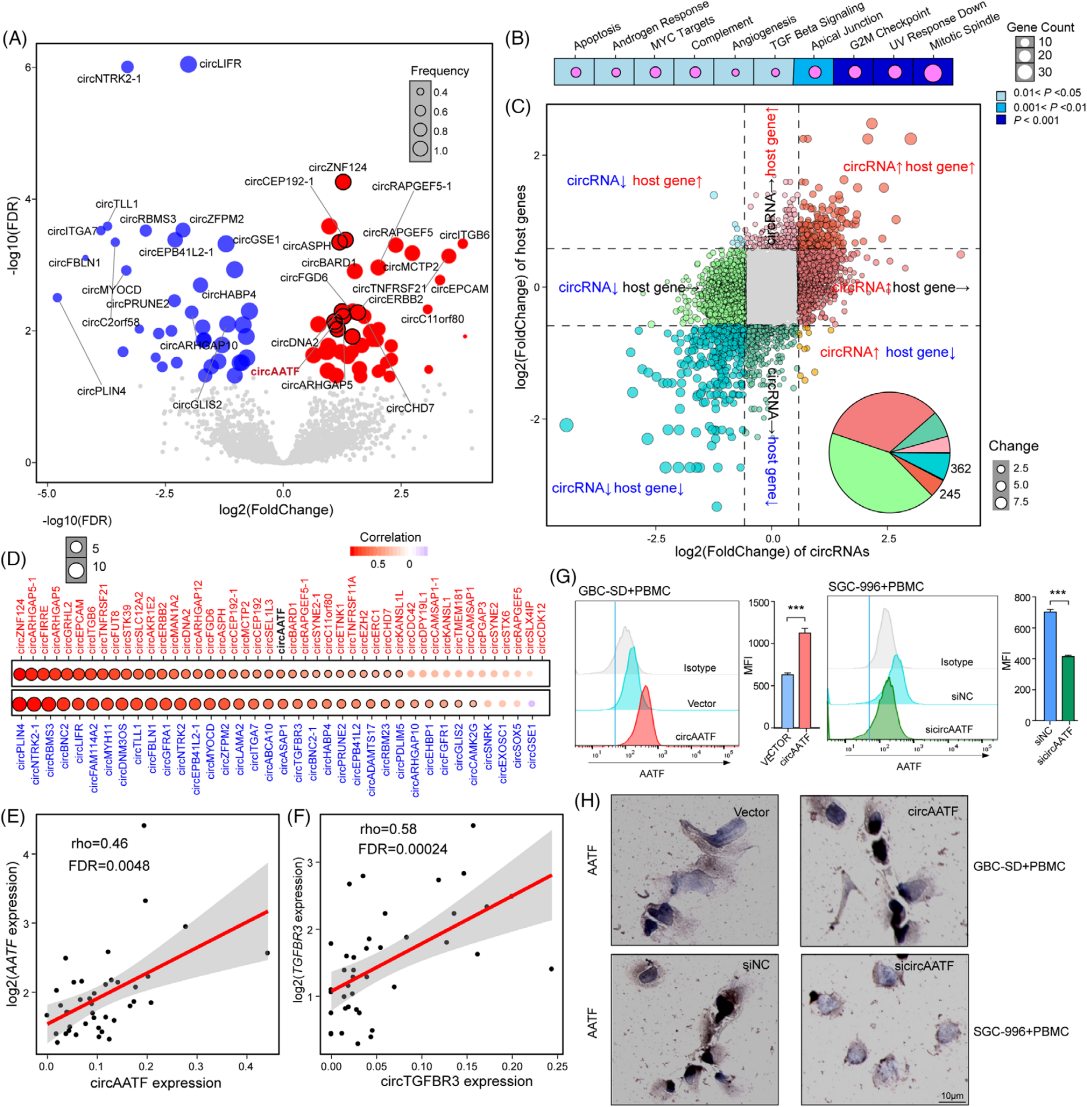
The expression dysregulation of circRNAs in GBC. (A) A volcano plot shows the difference in circRNA abundance between matched pairs of GBC tumour and NAT samples. (B) The top ten enriched Gene Ontology biological processes for genes expressing differential circRNAs. Circle size represents the number of involved genes, and background colours represent *p*‐values from the hypergeometric test. (C) The relationships between changes in circRNAs and their corresponding host genes. The scatter plot is segmented into eight distinct regions by dotted lines, corresponding to the expression changes of circRNAs and their host genes. Arrows positioned adjacent to the labels ‘circRNA’ and ‘host gene’ denote the nature of these changes. A black rightward arrow indicates no change in expression, a red upward arrow signifies upregulated expression, and a blue downward arrow represents downregulated expression. The inserted pie chart shows the percentage of different relationship types. (D) The expression correlations between differential circRNAs and their host genes. Circle size represents the –log10 transformed FDR value, and circle colour indicates the correlation coefficient. Red font indicates the upregulated circRNAs, and blue font indicates downregulated circRNAs. (E) Scatter plot showing the expression correlation between circAATF and *AATF*. (F) Scatter plot showing the expression correlation between circTFBR3 and TGFBR3. (G) Flow cytometry analysis of AATF expression in GBC‐SD+PBMC with circAATF OE, and in SGC‐996+PBMC with circAATF KD. Bar plots on the right panels show the MFI values. (H) IHC analysis of AATF expression in GBC‐SD+PBMC with circAATF OE and in SGC‐996+PBMC with circAATF KD. GBC‐SD+PBMC indicates GBC‐SD cells co‐cultured with PBMC, SGC‐996+PBMC signifies SGC‐996 cells co‐cultured with PBMC. Values are presented as mean ± SEM in (G). ^***^ *p* < .001.

To further examine the specific roles of circRNAs in GBC, we first identified differentially expressed circRNAs in GBC tumour samples compared to NAT samples (see Section 2). Consequently, 85 significantly differential circRNAs were identified (Table S3), including 47 upregulated and 38 downregulated circRNAs in GBC (Figure 2A). Functional enrichment analysis (see Section 2) revealed that dysregulated circRNAs were enriched in cell proliferation pathways such as ‘Mitotic spindle’, ‘G2 M checkpoint’, and ‘MYC targets’, genome instability pathways such as ‘UV response’, and tumour signalling such as ‘TGF beta signalling’ (Figure 2B). These observations suggest that differentially expressed circRNAs may participate in GBC tumour development. To explore the expression relationships between circRNAs and their corresponding parental genes in GBC, we correlated the expression changes of circRNAs with those of the parental genes (Figure 2C). Among these circRNAs showing expression changes, a total of 959 were upregulated, with 47 exhibited consistent expression changes with their parental genes (Figure 2D), accounting for 4.9%, such as circAATF (Figure 2E, rho = .46, FDR = .0048, Spearman's correlation). Additionally, 466 circRNAs were downregulated, with 34 showing consistent expression changes with their parental genes, accounting for 7.3%, such as circTGFBR3 (Figure 2F, rho = .58, FDR = .00024, Spearman's correlation). TGFBR3, a receptor for TGF‐β, is implicated in various cancers, including breast cancer, melanoma, prostate cancer, pancreatic cancer, colon cancer, multiple myeloma, neuroblastoma, ovarian cancer, endometrial cancer, and lung cancer.^34^, ^35^, ^36^, ^37^, ^38^ TGF‐β1 plays a pivotal role in gallbladder cancer progression through several mechanisms: it regulates IGFBP‐2, which facilitates malignant progression,^39^ enhances metastasis via m6A modification of FOXA1,^40^ and upregulates TUG1, thereby promoting cell proliferation and metastasis.^41^ While TGF‐β signalling has been extensively studied in various tumour contexts, research on AATF, particularly in the context of gallbladder cancer, remains limited. AATF, an apoptosis‐antagonising transcription factor, is crucial for tumour cell survival.^42^ Thus, further investigation into AATF is essential. In total, 85 circRNAs displayed consistent expression changes with their parental genes, representing 12.2%. AATF is an apoptosis‐antagonising transcription factor, which has been shown to be required for tumour cell survival.^42^ We next examined the levels of circAATF across several GBC cell lines, including GBC‐SD, NOZ, OCUG, SGC‐996 and G415 (Figure S3A). The GBC‐SD cell line displayed a relatively low baseline expression level of circAATF, while the SGC‐996 cell line showed a relatively high level of circAATF. Consequently, the GBC‐SD cell line was selected for circAATF overexpression (OE) experiments, and the SGC‐996 cell line for circAATF knockdown (KD) experiments. For circAATF knockdown, four regions surrounding the Alu elements, both upstream and downstream of the back‐splicing site of circAATF, were chosen as potential KD targets (Figure S3B). Two of these regions were found to effectively silence circAATF without affecting *AATF* mRNA levels and were ultimately selected as the targeting regions for circAATF KD (Figure S3C and D). We next performed flow cytometry analysis of *AATF* mRNA expression after circAATF OE in the GBC‐SD cell line and circAATF KD in the SGC‐996 cell line. Flow cytometry and IHC results indicated that the protein levels of AATF were significantly upregulated in GBC‐SD cells overexpressing circAATF, whereas they were markedly downregulated in SGC‐996 cells with circAATF KD (Figure 2G and H). These results indicate that dysregulation of circRNAs is associated with GBC tumour development, with circAATF elevating the expression level of linear AATF.

### 3.3 CircAATF promotes cell growth of GBC tumours

**FIGURE 3:**
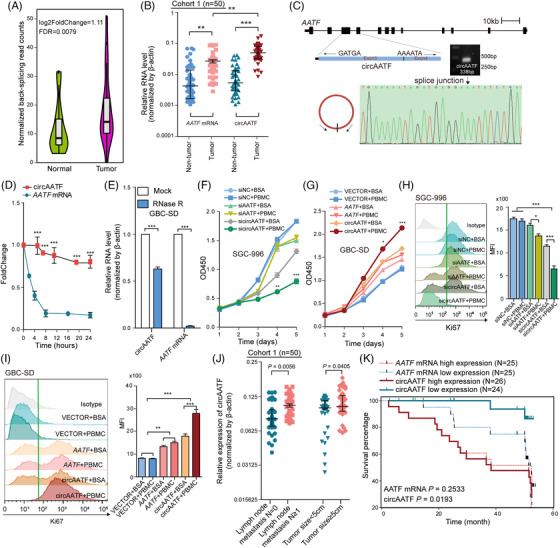
CircAATF promotes the proliferation of GBC cancer cells. (A) Comparison of circAATF expression levels between GBC tumour and NAT samples in RNA‐seq data. (B) Comparison of circAATF and *AATF* mRNA expression levels between GBC tumour and NAT samples in clinical samples. (C) Genomic location of circAATF. Abundance was quantified by RT‐PCR followed by Sanger sequencing. (D) Abundances of circAATF and *AATF* mRNA quantified by qRT‐PCR at different time points in GBC‐SD cells treated with Actinomycin D. (E) qRT‐PCR quantification of circAATF and *AATF* mRNA in GBC‐SD cells treated with RNase R. Abundances were normalised to the values measured in corresponding mock treatments. (F) Proliferation of GBC‐SD cells transfected with siNC, siAATF, and sicircAATF in BSA and PBMC culture, respectively. (G) Proliferation of SGC‐996 cells transfected with vector, *AATF*, and circAATF in BSA and PBMC culture, respectively. (H) Flow cytometry analysis of Ki‐67 expression in GBC‐SD cells with treatments: siNC+BSA, siNC+PBMC, siAATF+BSA, siAATF+PBMC, sicircAATF+PBMC, and sicircAATF+BSA. The bar plot on the left panel shows the relative quantity of expressed Ki67. (I) Flow cytometry analysis of Ki‐67 expression in SGC‐996 cells with treatments: vector+BSA, vector+PBMC, AATF+BSA, AATF+PBMC, circAATF+BSA, and circAATF+PBMC. The bar plot on the left panel shows the relative quantity of expressed Ki67. (J) Comparison of circAATF expression among groups with different clinicopathological features, including lymph node metastasis and tumour size. (K) Kaplan–Meier analysis of patient survival between groups with high and low circAATF expression, and high and low *AATF* mRNA expression. The difference test was performed using the log‐rank test. Values are presented as median with interquartile range in (A) and as mean ± SEM in (B) and (D‐I). ^*^ *p* < .05, ^**^ *p* < .01, ^***^ *p* < .001.

Next, the top differential circRNAs with a high sample frequency (>95% of samples) were selected to validate the circRNA transcripts by northern blotting assay (Figure S4A). The circular property was then confirmed in RNase R treated cells, where the selected circRNAs showed no significant expression changes compared to control cells (Figure S4B). Furthermore, the expression dysregulation of circAATF, circDNA2, circGSE1, and circASPH was examined in an independent GBC cohort (*N* = 15), where circAATF (chr17:36952885|36953907:+) exhibited the most significant dysregulation (Figure S4C). CircAATF demonstrated significant upregulation in our GBC RNA‐seq data (Figure 3A). We further investigated circAATF expression in an independent cohort, including 50 paired GBC tumour and NAT samples. The expression levels of circAATF and *AATF* mRNA were both significantly higher in GBC tumour samples compared to those in NAT samples (Figures 3B and S5A). The genomic structure indicated that circAATF was derived from the circularisation of exon 3 and 4 of the *AATF* gene, amplified by outward‐facing primers and further validated using Sanger sequencing (Figure 3C). The GBC‐SD cell line was utilised to examine the stability of circAATF. Specifically, total RNA samples were collected every 4 h after treatment with the transcription inhibitor, Actinomycin D. CircAATF showed stable transcript with a half‐life exceeding 24 h, while *AATF* mRNA exhibited a significantly shorter half‐life (less than 4 h) (Figure 3D). Additionally, GBC‐SD cells were treated with RNase R exonuclease, and the resistance to digestion confirmed the circular species of circAATF (Figure 3E). These results validated the stable circular property of circAATF in GBC. Quantification of circAATF in nuclear and cytoplasmic fractions revealed a preference for the cytoplasm, whereas *AATF* mRNA showed no difference (Figure S5B). Further fluorescence in situ hybridisation (FISH) confirmed that circAATF preferentially localised to the nuclei (Figure S5C). Our qRT‐PCR analysis indicated that circAatf was specifically expressed in mouse tissues such as the brain, lymph node, and spleen, with significantly higher abundance than linear *Aatf* mRNA (Figure S5D). CircAATF also showed ubiquitous expression across various cancer types, suggesting its potential roles in tumour (Figure S5E). We next explored the role of circAATF as an onco‐circRNA in GBC. Co‐culture of GBC cells and PBMCs in a simulated in vitro immune microenvironment was performed. After co‐culturing PBMCs with GBC cells for 48 h, we evaluated the impact of circAATF OE or KD on tumour growth. We observed that circAATF KD inhibited the proliferation of GBC‐SD cells (Figure 3F), while circAATF OE promoted the growth of SGC‐996 cells (Figure 3G). Moreover, circAATF KD significantly impaired the colony formation of GBC cells, whereas circAATF OE remarkably restored the colony formation (Figure S5F). Flow cytometry analysis of Ki67 further confirmed that circAATF KD inhibited GBC cell growth (Figure 3H), whereas circAATF OE was able to promote GBC cell proliferation (Figure 3I). These results suggest that the functional role of circAATF is primarily attributed to modulation of immune cell activity rather than exerting direct effects on GBC cell proliferation. This finding underscores the essential dependency of circAATF's functional role on the presence of the immune system. Additionally, circAATF was correlated with clinicopathological features of GBC patients, where high expression of circAATF was associated with lymph node metastasis and tumour size of GBC (Figure 3J and Table 1). Furthermore, higher circAATF expression was significantly associated with poorer prognosis of GBC patients, which was not observed for *AATF* mRNA (Figure 3K). Taken together, circAATF is able to promote the development of GBC tumours and is associated with the overall survival of GBC patients.

**TABLE 1:** Correlation of circAATF expression with clinicopathological characteristics in patients with GBC.

Characteristics	No. of patients	circAATF (low)	circAATF (High)	*χ* ^2^	*p* value
Gender				.0274	.8685
Male	32	15	17		
Female	18	8	10		
Age (year)				.1516	.6971
<60	16	8	8		
≥60	34	15	19		
TNM				5.3461	**.0208**
Early stage	12	9	3		
Late stage	38	14	24		
Tumour size (cm)				6.2691	**.0123**
< 5	17	12	5		
≥5	33	11	22		
Lymph node metastasis				8.8601	**.0029**
No	12	10	2		
≥1	38	13	25		
Tumour stage				13.3910	**.0003**
I–II	19	15	4		
III–IV	31	8	23		

*Note*: Low, mRNA levels in tumour tissues ≤ median value; High, mRNA levels in tumour tissues > median value. Bold fonts indicate significant *p* values.

### 3.4 CircAATF is positively associated with CD4^+^ T cells in GBC

**FIGURE 4:**
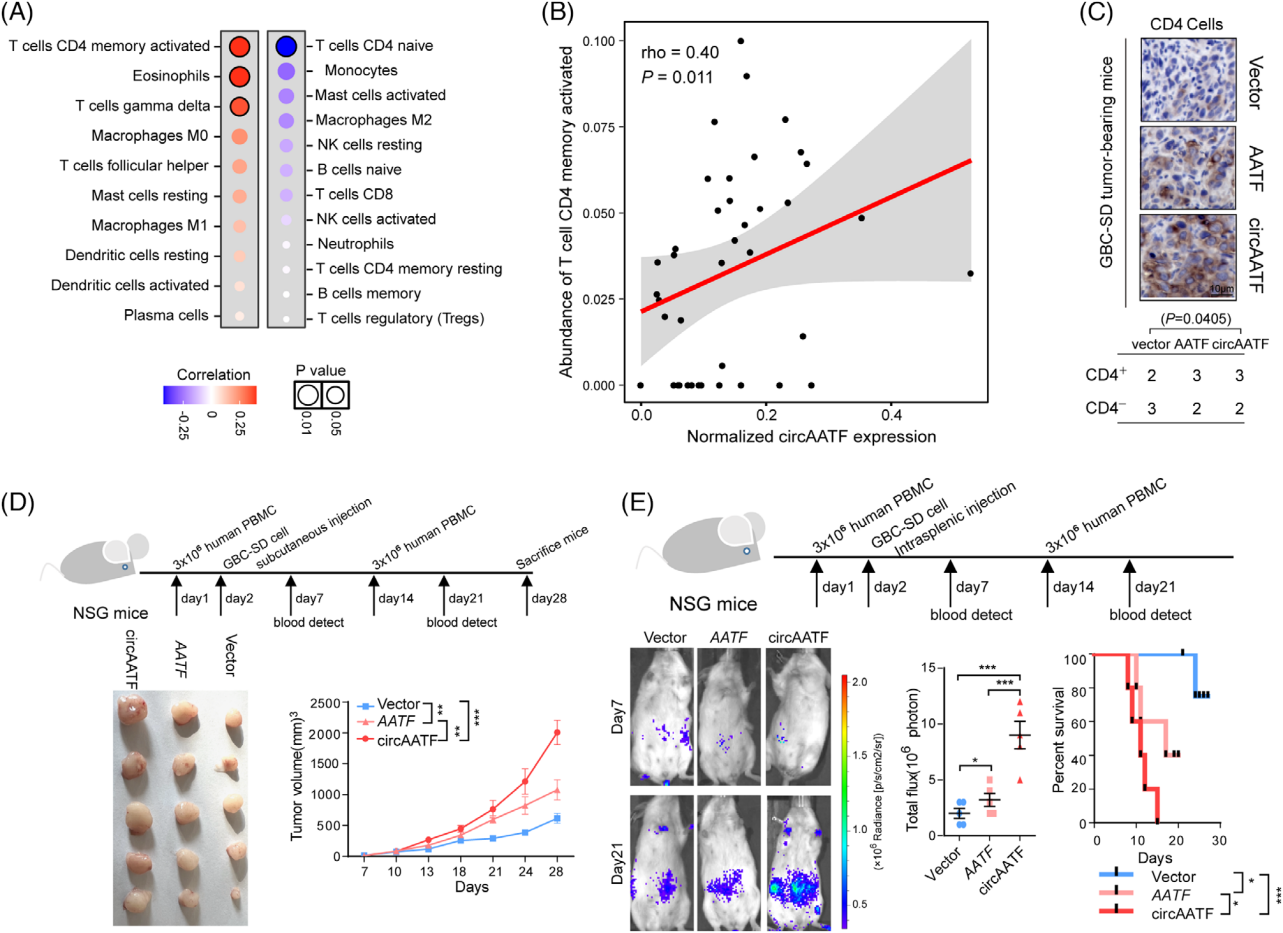
CircAATF is positively correlated with CD4^+^ T cells. (A) Correlations between differential circRNAs and various types of T cells. The upper panel, with red font, presents upregulated circRNAs, and the bottom panel, with blue font, presents downregulated circRNAs. (B) A scatter plot shows the positive correlation between circAATF expression and CD4^+^ T cell abundance in RNA‐seq data. (C) Representative immunohistochemistry (IHC) images show CD4^+^ memory activated T cell abundances in GBC tumours from different mouse groups. (D) Comparisons of tumour volumes among mouse groups injected with GBC‐SD cells transfected with vector, *AATF* mRNA, and circAATF. (E) Thermographic assessment of GBC tumours in mouse groups injected with GBC‐SD cells transfected with vector, *AATF* mRNA, and circAATF. Box plots in the middle panel show the total flux quantity in the corresponding groups. The right panel shows the comparison of survival times in different xenograft mouse groups. Values are indicated as mean ± SEM in (C‐E). ^*^ *p *< .05, ^**^ *p* < .01, ^***^ *p* < .001.

Since immune‐related hallmarks exhibited high‐level back‐splicing activity (Figure S2E), we next explored the involvement of circAATF in the tumour immunology of GBC. The correlations between the expression of circAATF and the abundances of various immune cell types were assessed (see Methods section in Supplementary Material). Our analysis revealed that circAATF was positively associated with ‘T cells CD4 memory activated’ and ‘Eosinophils’, while negatively correlated with ‘T cells CD4 naïve’ (Figure 4A). This observation suggested that circRNAs might be associated with activated T cells in GBC. Notably, circAATF expression showed a significantly positive correlation with CD4^+^ T cells (Figure 4B, rho = .40, *p* = .011, Spearman's correlation) in the RNA‐seq data. To further confirm the positive correlation between circAATF and CD4^+^ T cells, we developed a simulated microenvironment of human CD4^+^ T cell infiltration using NSG mice xenografts with intravenous injection of human PBMC to establish a humanised mouse model (see Section 2). We then injected the mice with GBC‐SD cells transfected with plasmids of circAATF, *AATF*, and control vectors. The mice were sacrificed 28 days after GBC cell injection. IHC analysis indicated that samples from circAATF‐injected mice contained the largest number of CD4^+^ T cells (Figure 4C), which was also observed for CD8^+^ T cells. As anticipated, mice transfected with circAATF developed the largest tumour sizes compared to those with *AATF* and control vector transfected mice (Figure 4D). Mouse weights showed no significant difference attributed to tumour growth among the different groups (Figure S6A). The promotion of GBC tumour growth by circAATF was further validated in mice injected with NOZ cells (Figure S6C and D). Mice with circAATF transfection exhibited significantly higher tumour growth than those with *AATF* and vector transfection under thermographic evaluation (Figure 4E). Moreover, a significantly shorter survival time was observed in mice with circAATF transfection compared to those with *AATF* and vector transfection in humanised xenograft mouse models (Figure 4E). The highest abundance of CD8^+^ T cells was also observed in mice transfected with circAATF (Figure S6B and E). These findings reveal that circAATF, an onco‐circRNA of GBC, is positively associated with CD4^+^ T cells in GBC.

### 3.5 CircAATF enhances anti‐PD‐L1 immunotherapy of GBC

**FIGURE 5:**
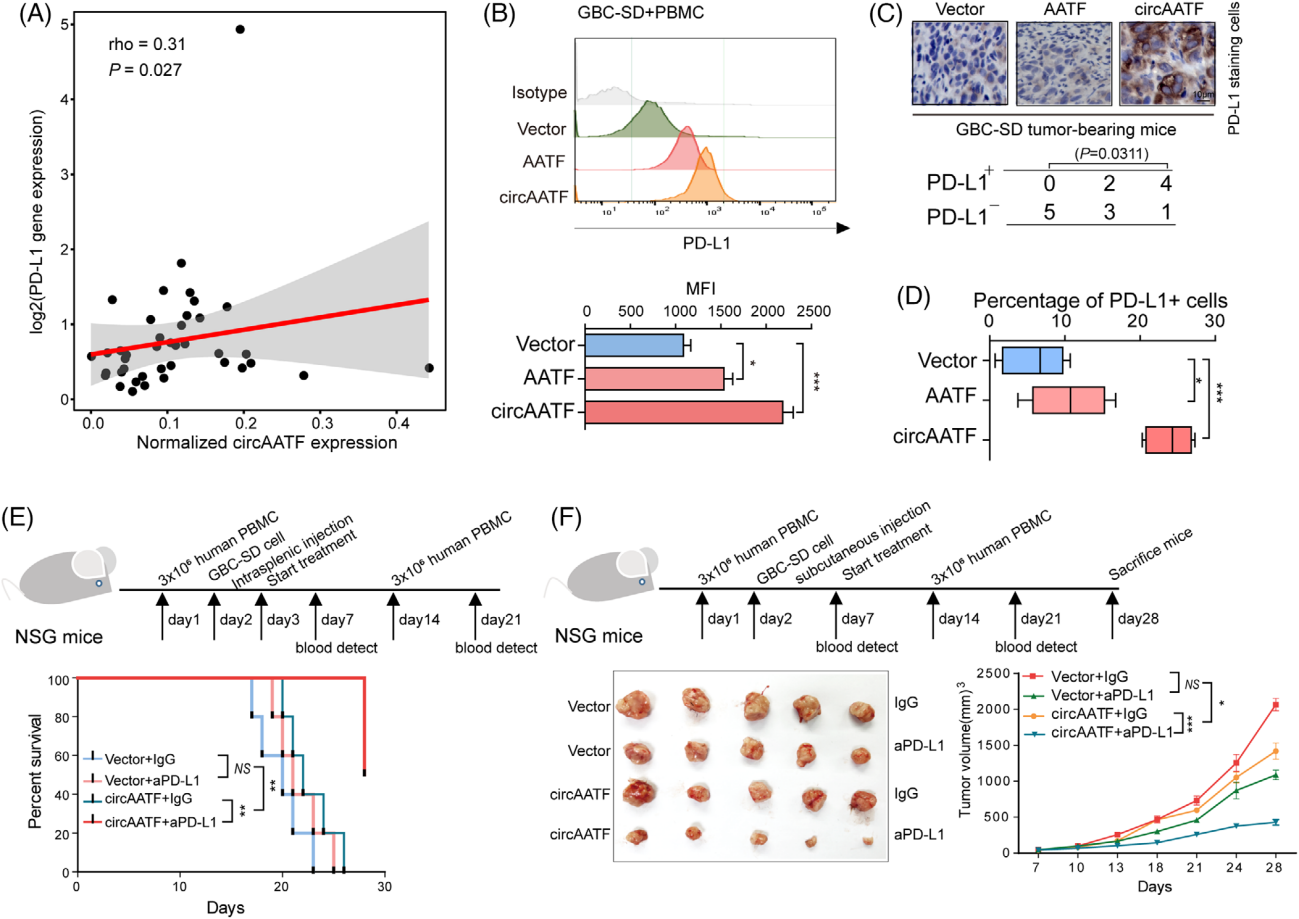
CircAATF enhances anti‐PD‐L1 treatment of GBC. (A) A scatter plot shows the positive correlation between circAATF and PD‐L1 gene expression. (B) Flow cytometry analysis of PD‐L1 expression in GBC‐SD+PBMC with vector, *AATF* mRNA, and circAATF OE. (C) Representative immunohistochemistry (IHC) images show PD‐L1 expression levels in GBC tumours from different mouse groups. (D) Comparison of the percentages of PD‐L1‐expressing cells among different mouse groups. (E) Survival analysis of mice treated with anti‐PD‐L1 and injected with GBC‐SD cells transfected with vector and circAATF. (F) Comparisons among mouse groups treated with anti‐PD‐L1 and injected with GBC‐SD cells transfected with vector or circAATF. Values are indicated as mean ± SEM in (C), (E), and (F). ^*^ *p* < .05, ^**^ *p* < .01, ^***^ *p* < .001.

To explore the clinical utility of circAATF in GBC, we further investigated whether circAATF was associated with PD‐L1, which binds to the inhibitory checkpoint molecule PD‐1 on CD4^+^ T cells and is widely targeted in ICB immunotherapy for solid tumours. Correlation analysis was performed between the expression of circAATF and the *CD274* gene (encoding the PD‐L1 protein) in GBC tumour samples. Our results showed that circAATF was significantly positively correlated with PD‐L1 (Figure 5A, rho = .31, *p* = .027, Spearman's correlation). Further flow cytometry analysis of PD‐L1 expression in PBMC co‐cultured with GBC‐SD cells confirmed that circAATF or *AATF* mRNA OE induced upregulation of PD‐L1 (Figure 5B). However, the upregulation of PD‐L1 was not induced by circAATF or *AATF* mRNA OE in BSA‐cultured GBC‐SD cells, determining that PBMCs are required to induce PD‐L1 expression for circAATF or *AATF* mRNA (Figure S7A and B). We then examined the PD‐L1 abundance in samples derived from GBC‐SD tumour‐bearing mice by IHC, where GBC‐SD tumours transfected with circAATF overexpressing plasmid showed the highest PD‐L1 expression (Figure 5C). Moreover, tumour samples from circAATF OE‐transfected mice exhibited the largest percentage of PD‐L1‐expressing cells (Figure 5D). Mice treated with anti‐PD‐L1 in the humanised xenograft model only showed a slight survival improvement compared to controls. Surprisingly, mice bearing circAATF transfected GBC‐SD tumours with anti‐PD‐L1 treatment showed the longest survival time (Figure 5E). The mice in the humanised mouse model were sacrificed on the 28^th^ day, and tumours were extracted and measured. Tumours derived from circAATF transfected mice with anti‐PD‐L1 treatment showed the smallest volume, indicating improved efficiency of anti‐PD‐L1 treatment in GBC with high circAATF expression (Figure 5F). Our results also showed that the suppression of tumour growth was not affected by the mice weights (Figure S7C). The enhancement of GBC anti‐PD‐L1 treatment by circAATF was also validated in mice with transfection of the NOZ cell line (Figure S7D and E). Taken together, circAATF was positively correlated with PD‐L1 expression in GBC and was shown to aid anti‐PD‐L1 immunotherapy for GBC.

### 3.6 CircAATF upregulates PD‐L1 through phosphorylated AKT and elevating *AATF* mRNA level

**FIGURE 6:**
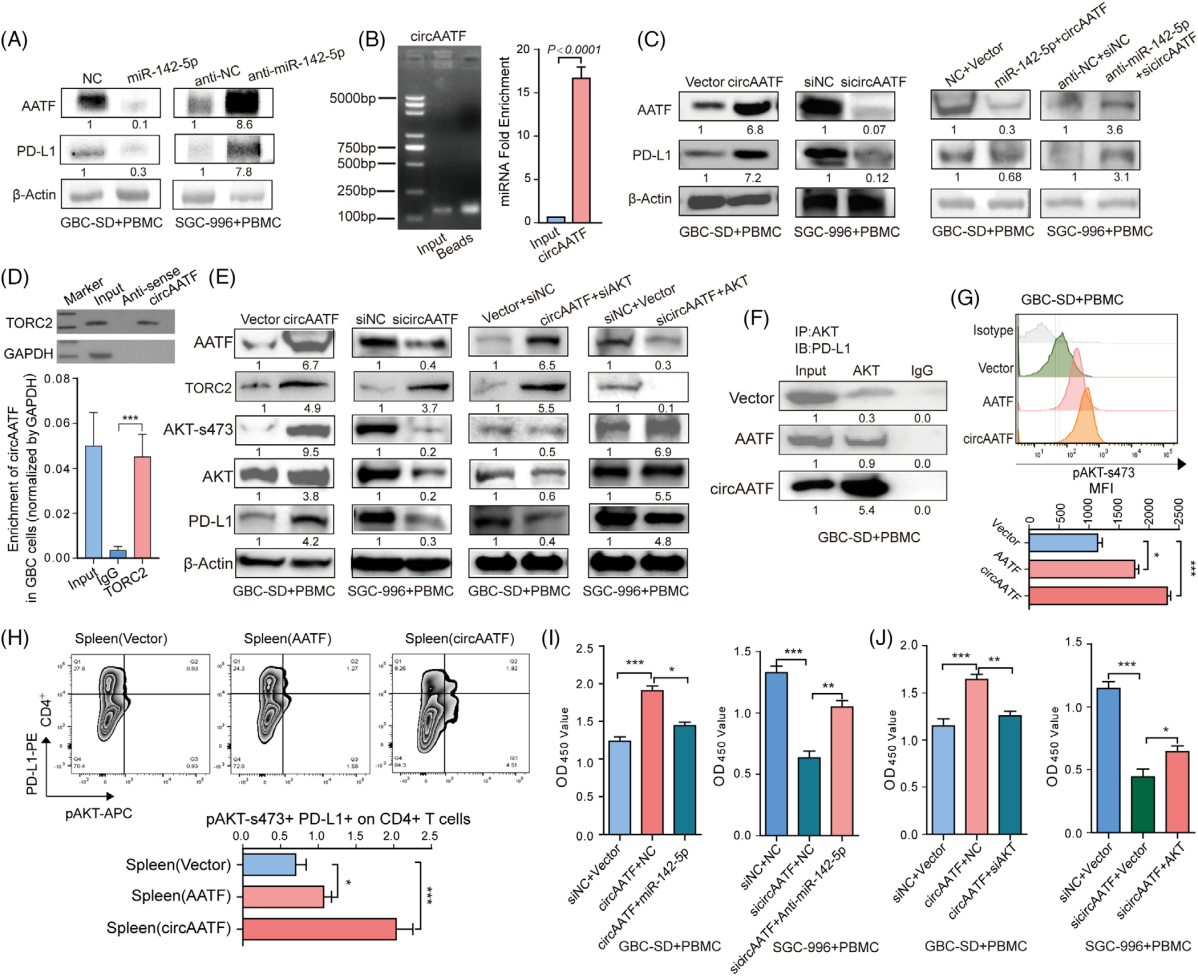
CircAATF upregulates PD‐L1 through phosphorylated AKT by elevating AATF mRNA level. (A) Western blotting results of AATF and PD‐L1 in cell lines and PBMC with miR‐142‐5p OE and KD. (B) Detection of circAATF in the miR‐142‐5p pull‐down assay. (C) Western blotting of circAATF OE and KD, combination of miR‐142‐5p and circAATF OE, and miR‐142‐5p and circAATF KD, respectively. (D) Co‐IP analyses show the interactions of TORC2 with circAATF, anti‐sense, and input. (E) GBC cells were transfected with circAATF, sicircAATF, circAATF+siAKT, sicircAATF+AKT, and the expression levels of AATF, TORC2, AKT‐s473, AKT, and PD‐L1 were determined by western blotting. (F) Immunoprecipitation assay shows that circAATF could promote the ability of AKT to bind directly to PD‐L1. (G) Following 48 h of co‐culture with PBMCs, flow cytometry analysis was conducted to evaluate *AATF* expression in GBC‐SD cells overexpression circAATF (GBC‐SD+PBMC with circAATF OE) and in SGC‐996 cells with circAATF knockdown (SGC‐996+PBMC with circAATF KD). The right panels present bar plots depicting the Mean Fluorescence Intensity (MFI) values. (H) After 48 h of co‐culture with GBC‐ PBMCs, IHC analysis was performed to assess *AATF* expression in GBC‐SD cells overexpressing circAATF (GBC‐SD+PBMC with circAATF OE) and in SGC‐996 cells with circAATF knockdown (SGC‐996+PBMC with circAATF KD). (I) Proliferation assays for GBC‐SD cells with circAATF+NC (negative control) and circAATF+miR‐142‐5p, and SGC‐996 cells with sicircAATF+NC and circAATF+anti‐miR‐142‐5p, respectively. (J) Proliferation assays for GBC‐SD cells with circAATF+NC and circAATF+siAKT, and SGC‐996 cells with sicircAATF+vector and sicircAATF+AKT, respectively. GBC‐SD+PBMC denotes GBC‐SD cells that have been co‐cultured with PBMCs, while SGC‐996+PBMC signifies SGC‐996 cells that have been co‐cultured with PBMCs. All western blot panels display greyscale values. In each experiment, the greyscale values of the internal reference were normalised to 1, and the greyscale values of the experimental groups were compared against this normalised internal reference. IP, immunoprecipitation. IB, immunoblotting. Values are indicated as mean ± SEM in (B) and (G)–(J). ^*^ *p* < .05, ^**^ *p* < .01, ^***^ *p* < .001.

We then explored the potential mechanism by which anti‐PD‐L1 immunotherapy was enhanced by circAATF in GBC. *AATF* mRNA and the PD‐L1 gene were found to share target regions of miR‐142‐5p in the 3′UTR (Figure S8A). Mutant 3′UTR of *AATF* and PD‐L1 were generated to examine the potential regulatory relationship between them. In both HEK‐293T and SGC‐996 cells, *AATF* and PD‐L1 with wide‐type 3′UTR exhibited coherently low expression, while those with mutant 3′UTR showed no expression changes compared to the control (Figure S8B). OE of miR‐142‐5p inhibited the expression of both *AATF* and PD‐L1, while levels of *AATF* and PD‐L1 were elevated after KD of miR‐142‐5p in PBMC co‐cultured GBC cells (Figure 6A). Utilising a bioinformatics prediction tool,^43^ we identified potential binding sites for miR‐142‐5p within the sequence of circAATF (Figure S8C). Subsequently, we employed a dual‐luciferase reporter assay system to validate the interaction between circAATF, encompassing exons 3 and 4 of the *AATF* mRNA, and miR‐142‐5p. Our experimental results confirmed a specific interaction between circAATF and miR‐142‐5p (Figure S8D). Furthermore, through FISH experiments, we verified the nuclear localisation of miR‐142‐5p in the SGC‐996 cells (Figure S8E), demonstrating its presence within the nucleus. Furthermore, we confirmed that circAATF could sponge miR‐142‐5p in SGC‐996 cells (Figures 6B and S8F). Consequently, circAATF OE resulted in higher expression of *AATF* and PD‐L1, whereas KD of circAATF suppressed both *AATF* and PD‐L1 in PBMC co‐cultured GBC cells (Figure 6C). Additionally, *AATF* and PD‐L1 showed a much smaller extent of expression changes with OE or KD of both circAATF and miR‐142‐5p in PBMC co‐cultured GBC cells, indicating that circAATF might reduce the impact of miR‐142‐5p on the expression of *AATF* and PD‐L1. These findings suggested that circAATF might regulate PD‐L1 through modulating *AATF* expression, wherein *AATF* regulated PD‐L1 levels by miR‐142‐5p. To investigate whether circAATF functions through interactions with proteins, we performed pull‐down assays on circAATF. The pull‐down assay revealed the interaction between circAATF and the TORC2 protein (Figures 6D and S8G). Furthermore, circAATF OE elevated AKT‐s473 levels, while AKT‐s473 levels decreased after circAATF KD (Figure 6E). However, circAATF OE combined with AKT KD or circAATF KD combined with AKT OE exhibited no significant impact on PD‐L1 levels, compared to the vector groups. AKT OE or KD reversed the effects of circAATF KD or circAATF OE on PD‐L1 levels, respectively. We further found that AKT OE GBC‐SD cells co‐transfected with circAATF exhibited much higher PD‐L1 expression levels than those transfected with *AATF* in PBMC co‐cultured GBC cells (Figure 6F). Furthermore, flow cytometry analysis of pAKT‐s473 levels showed that circAATF OE induced much higher levels of pAKT‐s473 than *AATF* mRNA and control (Figure 6G). This was observed in spleen CD4^+^ T cells and CD8^+^ T cells as well (Figures 6H and S8H). These results indicate that the regulatory role of circAATF might be exerted through phosphorylated AKT‐s473. In addition, circAATF OE combined with miR‐142‐5p OE showed significantly less effect on promoting GBC cell growth, whereas circAATF KD combined with miR‐142‐5p KD exhibited low impact on inhibiting GBC cell proliferation under co‐culture with PBMCs (Figure 6I). Moreover, circAATF OE combined with AKT KD exhibited significantly less effect on promoting GBC cell proliferation, while circAATF KD combined with AKT OE showed significantly less effect on suppressing GBC cell growth under co‐culture with PBMCs (Figure 6J). Our analysis revealed that circAATF upregulated PD‐L1 expression at the transcriptional level via activating AKT signalling, and at the post‐transcriptional level via eliminating the inhibitory effects of miR‐142‐5p, thus leading to a tumour immune evasion mode; the miRNA sponge effects of circAATF also led to an elevated level of linear *AATF* expression, which directly promoted the cell growth of GBC (Figure S9).

## 4 DISCUSSION

Although recent studies have reported genomic profiling of GBC,^44^, ^45^, ^46^ the primary molecular triggers of GBC remain to be elucidated. The transcriptomic landscape of GBC, particularly that of low‐abundance and non‐poly(A) transcripts, has not yet been characterised. By utilising ultra‐deep rRNA‐depleted RNA sequencing of GBC samples, we presented a comprehensive characterisation of CircRNome in GBC, which supplements the molecular characterisation of GBC. For circRNA identification, combining various tools improves reliable and balanced performance.^47^ We employed four popular circRNA identification tools for parallel identification, then retained circRNAs detected by at least two tools. Furthermore, we filtered the circRNAs to remain only the high‐confidence GBC circRNAs that had at least two sequencing reads covering back‐splicing sites in at least five GBC tumour or NAT samples. More than 90% of GBC high‐confidence circRNAs were found in the MiOnCirc database. Therefore, we present the most reliable and comprehensive GBC circRNA catalogue to date, which will contribute significantly to circRNA research.

The *BIRC6* gene was found to express the largest number of circRNAs in GBC samples, which has also been observed in other cancer types.^9^ *BIRC6* encodes a protein with a BIR (baculoviral inhibition of apoptosis protein repeat) domain and a UBCc (ubiquitin‐conjugating enzyme) domain. It inhibits apoptosis by promoting the ubiquitination and degradation of apoptotic proteins. BIRC6‐generated circRNAs have been reported to play roles in various human cancers, including hepatocellular carcinoma^48^ and non‐small cell lung cancer.^49^ However, BIRC6‐derived circRNAs did not exhibit significantly differential expression in GBC. Thus, these circRNAs were not further investigated in the present study.

In the comparative analysis of expression changes between circRNAs and their parental genes, we found that a subset of circRNAs exhibited inverse changes compared to their parental genes in GBC tumour samples. Previous studies have demonstrated that circRNAs can serve as ‘mRNA traps’ to inhibit the expression of parental genes.^50^ Specifically, when a circRNA encompasses the translation start site of its parental gene, it may act as an mRNA trap by sequestering the translation start site.^51^, ^52^, ^53^ Consequently, these circRNAs might function as ‘mRNA trap’ to suppress the expression of their parental genes. This inconsistent expression change suggests distinct clinical roles of circRNA in GBC. In our differential expression analysis, we identified 47 upregulated and 38 downregulated circRNAs in GBC tumour samples, which could potentially serve as indicators to distinguish GBC tumour samples from normal gallbladder samples. The expression levels of circRNAs vary across tumour samples,^9^, ^20^ a phenomenon also observed in our GBC samples. Those circRNAs that are highly expressed in certain cancer samples could serve as markers to distinguish subtypes of GBC cancer samples. In the prognosis analysis, we observed that the expression level of the host gene *AATF* was not significantly associated with patient survival but followed the same trend as circAATF. Given that the expression level of *AATF* is positively correlated with that of circAATF, we hypothesise that *AATF* may be significant for prognosis prediction in a larger cohort.

The humanised xenograft mouse model used in this study has limitations, such as the mismatch of human leukocyte antigens (HLA) types between GBC‐SD tumour cells and human PMBCs. We employed reasonable control groups; the reactions in these control groups throughout the study can be used to elucidate the biological function of circAATF. In addition, T cells are selected in the mouse thymus and remain ‘xeno’ to the tumour cells, even if the HLA types are completely matched.^54^ Furthermore, the degree of HLA matching in the humanised NSG model has been found to have no significant impact on immune response and immunotherapy.^55^ Thus, the humanised xenograft mouse model is effective in this study. Nevertheless, we are still endeavouring to construct a humanised mouse model of GBC with complete HLA types.

Immunotherapy is emerging as a crucial approach in cancer treatment, offering new avenues for systematic therapy in biliary tract cancer (BTC) and other malignancies. Increasing evidence from clinical trials indicates that immunotherapy is effective for BTC, suggesting its inclusion in standard treatment regimens.^5^ However, currently only a small proportion of BTC patients respond to immunotherapy, and some immune drugs alone have limited efficacy.^5^ Therefore, further research into combination treatment strategies is necessary to provide new therapeutic options for advanced GBC patients. Our analysis found that the expression level of circAATF was significantly positively correlated with that of *AATF* mRNA. Through comparative sequence analyses, we revealed that *AATF* mRNA and the PD‐L1 gene shared the target region of miR‐142‐5p in the 3′ UTR. Through the pull‐down assay, we found that the TORC2 protein binds to circAATF. The TORC2 protein is well‐known for its important role in the S473 phosphorylation of AKT.^56^, ^57^, ^58^ Additionally, AKT has been demonstrated to tightly regulate the expression of PD‐L1.^59^ Thus, we further investigated the impact of circAATF on AKT phosphorylation and activation. Our study demonstrated that circAATF upregulates PD‐L1 expression through AKT phosphorylation and by elevating linear *AATF* expression, which in turn regulates PD‐L1 expression through miR‐142‐5p. CircAATF might activate pAKT by binding to TORC2. Our findings suggest that circAATF plays a crucial role in mediating the immune escape of tumour cells in bile duct cancer and could serve as an effective biomarker for predicting immunotherapy outcomes. Our animal experiments confirmed that circAATF enhances sensitivity to PD‐L1 antibody treatment, highlighting its potential to aid PD‐L1 immune therapy for GBC. CircAATF not only plays a key role in the immune escape mechanisms of bile duct cancer but may also serve as an important biomarker for predicting the efficacy of immunotherapy, providing a theoretical foundation for developing novel therapeutic strategies.

In current study, we employed a widely used computational algorithm to estimate the abundance of T cells in GBC samples. Although the estimation might have been affected by the heterogeneity of tumour samples, we validated our results using experimental approaches in both GBC cell lines and humanised xenograft mouse models. We also found that GBC xenograft mice with high levels of circAATF responded favourably to anti‐PD‐L1 treatment. Recent efforts have deciphered therapeutic options for GBC patients.^44^, ^45^, ^46^ For example, a multi‐omics analysis of GBC samples revealed frame‐shift mutations in *ELF3* that generated cancer‐specific neoantigens activating T cells, which are promising candidates for cancer vaccine.^46^ The potential roles of circRNAs in the regulation of immunology have been suggested,^15^, ^23^, ^60^ implicating their promising utilisation in cancer immunotherapy.^61^ For instance, Cai et al. found that the suppression of circRHBDD1 may enhance the efficacy of anti‐PD‐1 therapy for hepatocellular carcinoma treatment.^62^ Despite the preliminary results in the GBC xenograft mouse model, our study indicates that circAATF may serve as a promising biomarker and candidate for combination strategies in GBC immunotherapy. A large number of patient data for immunological investigation will facilitate the clinical translation of our findings in GBC.

## AUTHOR CONTRIBUTIONS

S.L., J.Z., and H.L. conceived and designed the project. Y.W., X.B., and C.W. collected and managed the clinical samples. S.L., Y.W., and X.B. performed the computational analyses. Y.W., C.W., Y.L., and L.N. conducted and analysed the primary cell experiments. J.Z., Y.L., D.Z., and Y.W. carried out the animal experiments. S.L., J.Z., and H.L. drafted the manuscript with input from all authors. All listed authors read and approved the final manuscript.

## CONFLICT OF INTEREST STATEMENT

The authors declare no potential conflicts of interest.

## ETHICS STATEMENT

All experimental methods applied in this study are in compliance with the Declaration of Helsinki. The human cancer cell lines used in this study were obtained from ATCC. All animal experiments were conducted following the Guide for the Care and Use of Laboratory Animals. All work involving human tissues was performed with written informed consent from patients at Fudan University Zhongshan Hospital, under an institutional review board‐approval protocol.

## Supporting information



Supporting information

Supporting information

Supporting information

## Data Availability

The raw sequencing reads have been deposited in the Gene Expression Omnibus (GEO, http://www.ncbi.nlm.nih.gov/geo/) database, under the accession code GSE138109. The software and resources used in this study are detailed in the respective methods section.
