# Supplementary material for: CircRNome‐wide characterisation reveals the promoting role of circAATF in anti‐PD‐L1 immunotherapy of gallbladder carcinoma
Source: Clin Transl Med. 2024 Oct 20;14(10):e70060. doi: 10.1002/ctm2.70060 (PMC11491271; doi:10.1002/ctm2.70060)
Supplement: Supplementary file 1 — Supporting information [file CTM2-14-e70060-s003.pdf]

Supplementary Tables

Supplementary Table S1. Sequencing statistics of RNA-seq library in each sample.

Sample	Total read count	Mapped read count
GBCN1	168,069,822	164,333,683
GBCN2	191,620,521	187,293,459
GBCN3	160,119,410	156,654,279
GBCN4	157,183,998	153,525,510
GBCN5	109,498,556	106,613,581
GBCN6	152,812,898	149,426,945
GBCN7	164,325,015	159,719,069
GBCN8	158,155,886	154,269,743
GBCN9	142,017,196	139,014,837
GBCN10	169,052,196	163,899,101
GBCN11	163,379,681	159,321,223
GBCN12	145,119,434	142,158,894
GBCN13	164,111,751	159,939,500
GBCN14	146,257,532	139,775,590
GBCN15	116,207,655	113,269,179
GBCN16	116,917,411	114,672,775
GBCN17	140,974,809	136,681,443
GBCN18	115,180,208	112,937,060
GBCN19	126,235,426	123,332,063
GBCN20	146,957,008	143,404,721
GBCT1	188,381,174	183,823,899
GBCT2	177,342,842	174,361,025
GBCT3	155,872,625	151,553,126
GBCT4	177,321,412	170,371,240
GBCT5	164,743,087	159,991,833
GBCT6	143,566,620	140,164,404
GBCT7	167,168,486	162,688,650
GBCT8	137,757,332	134,703,807
GBCT9	204,182,112	199,150,250
GBCT10	131,213,137	128,164,374
GBCT11	168,155,473	162,701,796
GBCT12	123,324,419	114,791,619
GBCT13	153,358,214	150,222,334
GBCT14	154,364,775	150,363,414
GBCT15	127,377,015	124,598,030
GBCT16	126,635,715	123,949,125
GBCT17	136,038,550	132,717,793
GBCT18	118,877,198	116,412,649
GBCT19	123,406,368	121,155,992
GBCT20	136,805,141	133,826,818

Supplementary Table S2. CircRNAs identified in GBC samples.

Supplementary Table S3. Differential circRNAs in GBC tumor samples.

Supplementary Table S4. Knockdown primers.

RNAs	Primer sequences
circAATF-F	ACCACAGGCAAGCTAATCCTCCA
circAATF-R	GGCTAATGGTGTTTCAGATAGGA
AATF-F	TCTACAGGAACCGCACACTTCAGAAA
AATF-R	TCACTGGTTTCCTCCATCTGGACCAGC
ACTIN-F	CTCCATCCTGGCCTCGCTGT
ACTIN-R	GCTGTCACCTTCACCGTTCC
gRNA1	CCACAGTGCTGAAAAGAAGAAA
gRNA2	AAAGCACAATAAACATGGCTCA
gRNA3	ACCCATTTTTCTCTTTCTTCCC
gRNA4	GGGAAGAAAGAGAAAAATGGGT
gRNA5	AAGCACAATAAACATGGCTCAA
gRNA6	ATGCCACAGTGCTTAAGTTGAA
circAATF	GAAAAATAGATGAGGAA
AATF	GAGAGCAAGAAGAGCAGAA

Supplementary Table S5. Primers used for circRNAs.

circRNA	Primer sequences
circMPP6-F	CAAGGTGTAGGCCGAAGAA
circMPP6-R	GGCAGCTCCGTAAGGTTTT
circRASA3-F	CAGAAGGAGGACTTGCAGAA
circRASA3-R	CGGGTAAGAGGGAAGGTTT
circXPO1-F	CCAAGGAACCAGTGCGAAG
circXPO1-R	GAAATCAAGCAGCTGACGA
circPVT1-F	TTCAGCACTCTGGACGGACTT
circPVT1-R	TATGGCATGGGCAGGGTAG
circHIPK3-F	TATGTTGGTGGATCCTGTTCGGCA
circHIPK3-R	TGGTGGGTAGACCAAGACTTGTGA
circEZH2-F	TGGAAACAGCGAAGGATAC
circEZH2-R	TCGTCTGAACCTCTTGAGC
circUBAP2-F	GAGTTTGGGCCAGTTTACC
circUBAP2-R	GAGCAGATGAGGCAGTGAA
circATXN1-F	GATTGAAGACAGCCATAGCC
circATXN1-R	CTGATAAACGGAAAGTCACATT
circDHRS3-F	CCAGCACCGAGATGTTCCA
circDHRS3-R	TCATCACTGTCCATTAGGCTCTT
circRTN4-F	AGTAATTCTGCTCTTGGTC
circRTN4-R	GTCTTCTTAATGTCTCTCC
circFOXK2-F	TAATGGCTGACAACTCACA
circFOXK2-R	GCTTCTCTCTCTTCTCGCT
circDNA2-F	GGCTAAGATCAAACCTACTCCCT
circDNA2-R	ACGTTTACATGTAGGAACCTAAGC
circASPH-F	TGAGAACCACTGAAAGGCTATAGAA
circASPH-R	CATTTGACACTGCTTTGCAGGA
circGSE1-F	TCGCTAGGGATGCTTTCCAC
circGSE1-R	GTGAGGGGGTTGACGGTG
GAPDH-qF	GTCAAGGCTGAGAACGGGAA
GAPDH-qR	AAATGAGCCCCAGCCTTCTC
Actin-qF	TTGTTACAGGAAGTCCCTTGCC
Actin-qR	ATGCTATCACCTCCCCTGTGTG
18S-qF	TTAATTCCGATAACGAACGAGA
18S-qR	CGCTGAGCCAGTCAGTGTAG

Supplementary Figures

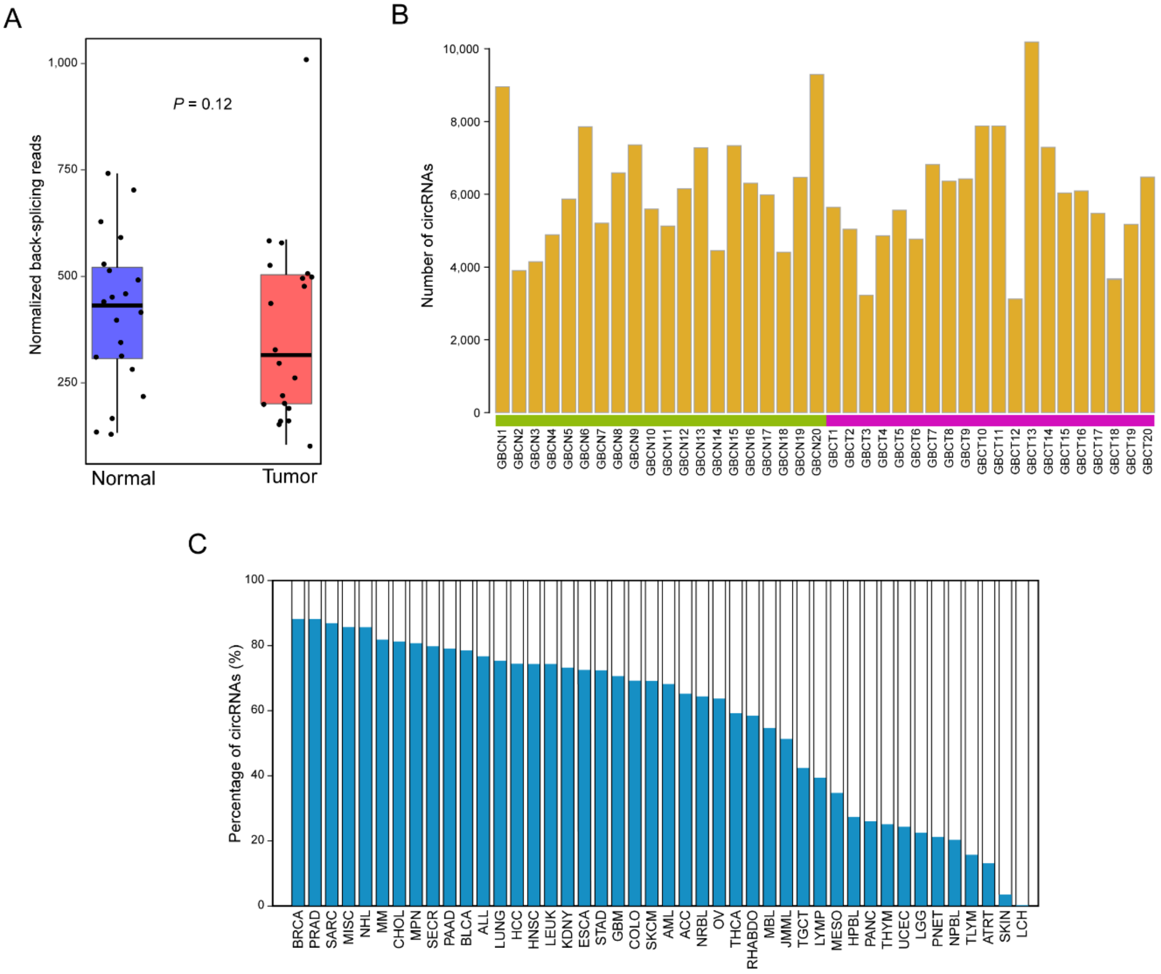

Supplementary Figure S1. Distribution of back-splicing events in GBC samples. (**A**) The distribution of back-splicing reads (normalized by the total sequencing reads in each sample) in GBC tumor and normal adjacent tissue (NAT) samples. (**B**) The number of circRNAs in each sample. (**C**) The percentage of detected circRNAs in each cancer type from the MiOncoCirc database. BRCA: breast invasive carcinoma, PRAD: prostate adenocarcinoma, SARC: sarcoma, MISC: miscellaneous, NHL: non-Hodgkin lymphoma, MM: multiple myeloma, CHOL: cholangiocarcinoma, MPN: myeloproliferative neoplasm, SECR: glandular cancer, PAAD: pancreatic cancer, BLCA: bladder cancer, ALL: acute lymphoblastic leukemia, LUNG: lung cancer, HCC: hepatocellular carcinoma, HNSC: head and neck cancer, LEUK: leukemia, KDNY: kidney, ESCA: esophageal cancer, STAD: stomach cancer, GBM: glioblastoma multiforme, COLO: colon cancer, SKCM: skin cutaneous melanoma, AML: acute myeloid leukemia, ACC: adrenal carcinoma, NRBL: neuroblastoma, OV: ovarian cancer, THCA: thyroid carcinoma, RHABDO: rhabdomyosarcoma, MBL: monoclonal B-cell lymphocytosis, JMML: Juvenile myelomonocytic leukemia, TGCT: testicular germ cell tumors, LYMP: lymphoma, MESO: mesothelioma, HPBL: hepatoblastomas of childhood, PANC: pancreatic cancer, THYM: thymoma, UCEC: uterine corpus endometrial carcinoma, LGG: brain lower grade glioma,

PNET: primitive neuroectodermal tumor, NPBL: non-palpable breast lesions, TLYM: testicular lymphoma, ATRT: atypical teratoid rhabdoid tumor, SKIN: skin cancer, LCH: Langerhans cell histiocytosis.

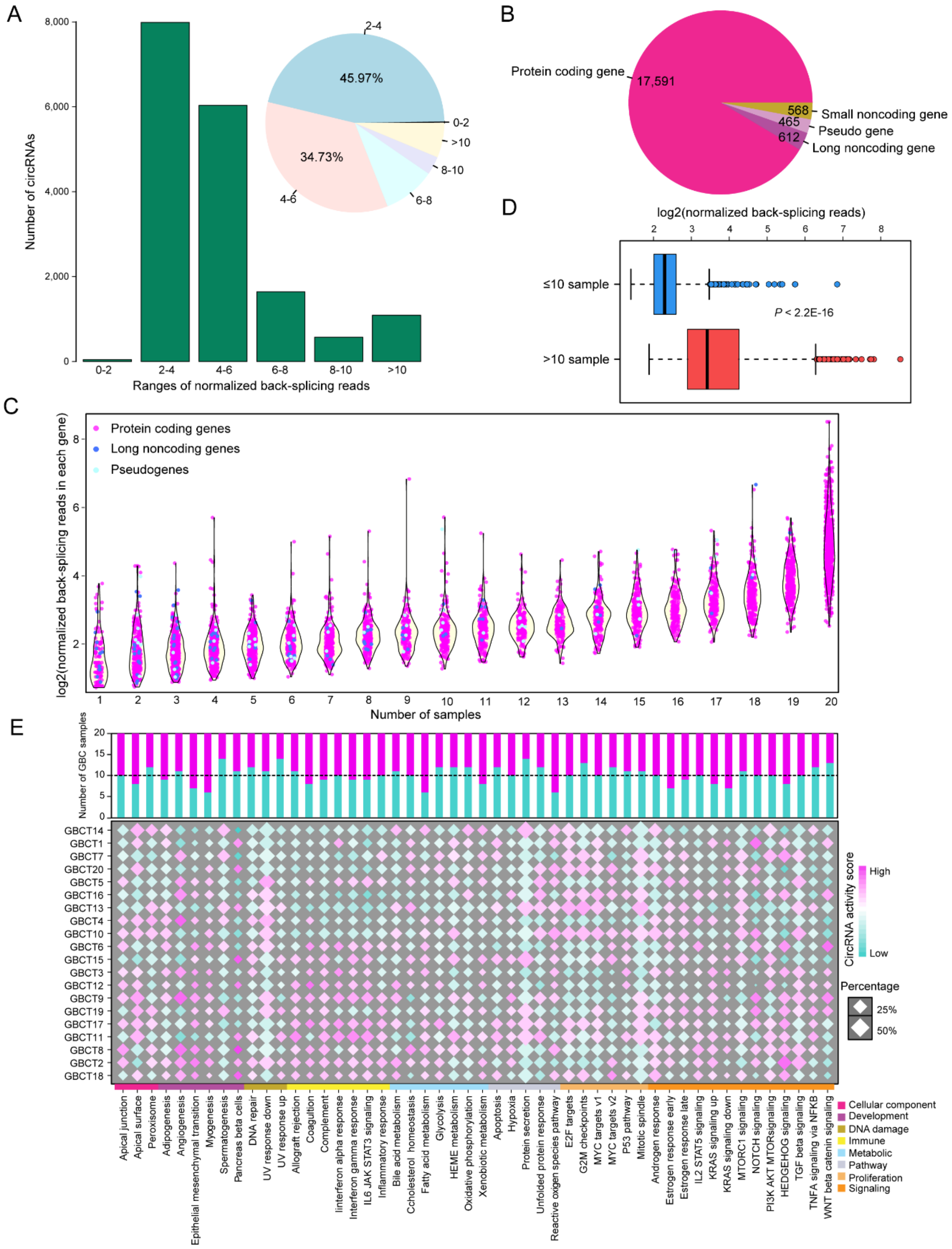

Supplementary Figure S2. Abundance and sample frequency of GBC circRNAs. (**A**) The distribution of circRNA expression levels in GBC. (**B**) A pie chart shows the percentage of gene types that generate circRNAs. (**C**) The distribution of back-splicing reads generated from different types of genes across various sample frequencies. (**D**)

Comparison of back-splicing reads between circRNAs detected in more than 10 GBC samples and those in 10 or fewer samples. The p-value was generated from Wilcoxon rank sum test. (**E**) CircRNA activity scores in differential biological hallmarks. Bar plots in the upper panel show the number of corresponding GBC samples. Rhombus color indicates the level of circRNA activity scores, and rhombus size indicates the percentage of genes generating circRNAs.

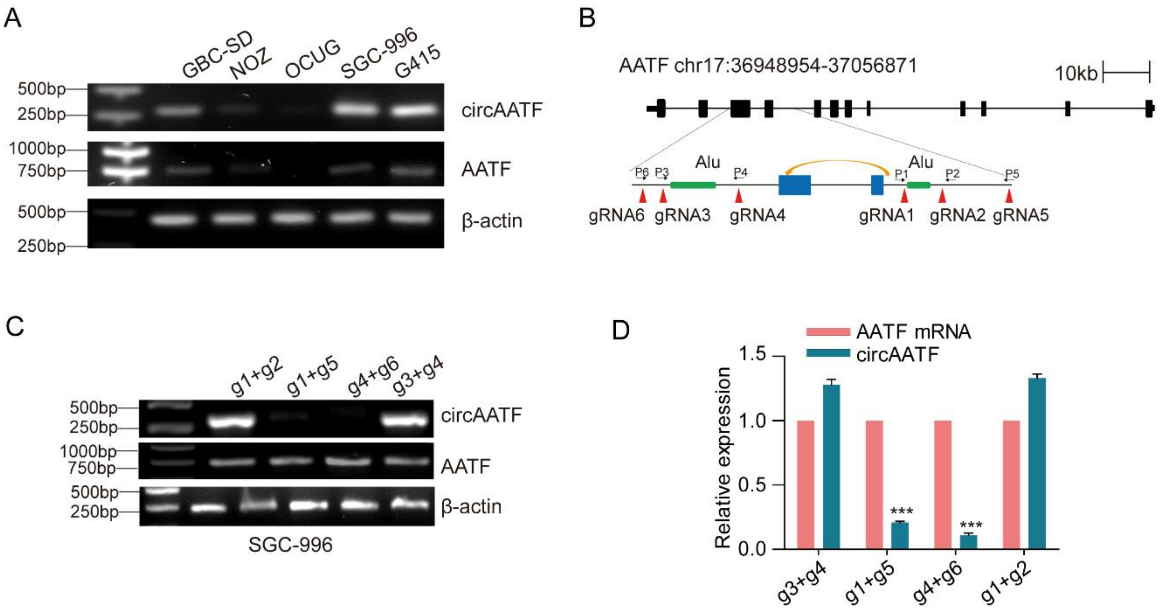

Supplementary Figure S3. Knockdown of circAATF in GBC cells targeted to various regions. (**A**) Levels of circAATF in different GBC cell lines quantified by northern blotting. (**B**) Genomic locations of the selected silencing targets around Alu elements near circAATF. (**C**) Northern blotting analysis showing circAATF and *AATF* mRNA levels after silencing different target regions of circAATF in the SGC-996 cell line. (**D**) Relative expression of circAATF and *AATF* mRNA after silencing different target regions of circAATF. $^{***}P < 0.001$

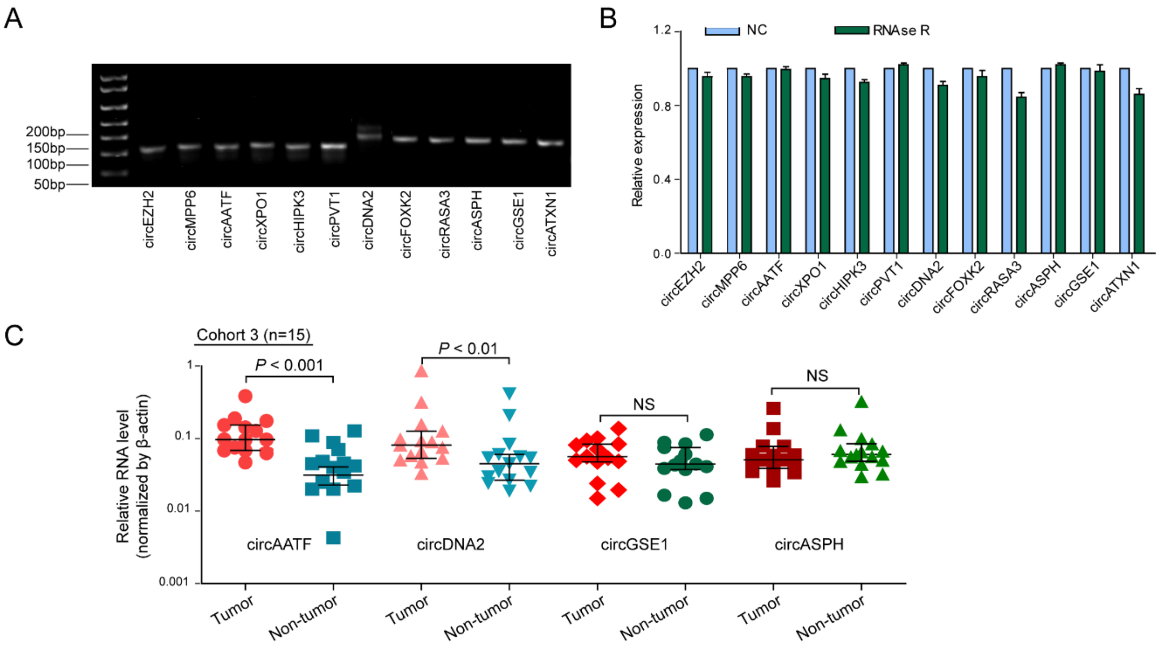

Supplementary Figure S4. Expression levels of selected top differential circRNAs in independent GBC cohorts. (**A**) Northern blotting of selected top differential circRNAs, including circEZH2, circMPP6, circAATF, circXPO1, circHIPK3, circPVT1, circDNA2, circFOXK2, circRASA3, circASPH, circGSE1, and circATXN1. (**B**) Relative expression levels of selected top differential circRNAs in RNAse R-treated and control cell lines. (**C**) Comparison of expression levels of selected differential circRNAs in paired GBC tumor and NAT samples. Values are presented as median with interquartile range in (**B**) and as mean ± SEM in (**C**).

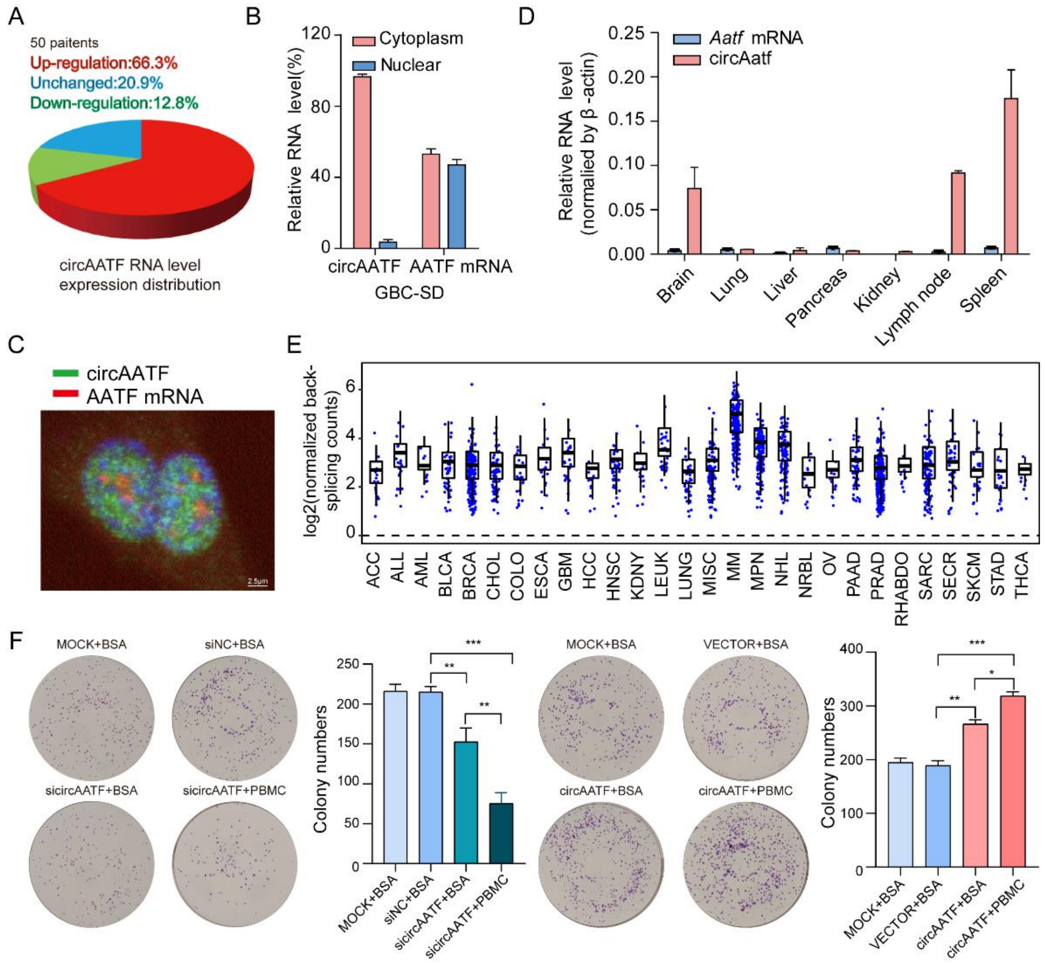

Supplementary Figure S5. Dysregulation and functions of circAATF in GBC. (**A**) The distribution of circAATF expression changes across 50 paired GBC clinical samples. (**B**) Levels of circAATF and *AATF* mRNA in the cytoplasm and nucleus of GBC-SD quantified by qRT-PCR. (**C**) RNA fluorescence *in situ* hybridization for circAATF and *AATF* mRNA. (**D**) Relative RNA levels of circAatf and *Aatf* mRNA across different tissue types in Balb/c mice. (**E**) Expression of circAATF and *AATF* mRNA across various cancer types derived from the MiOncoCirc database. (**F**) Rescue assays of colony formation following circAATF silencing, overexpression of circAATF, and their respective controls. $^{*}P < 0.05$, $^{**}P < 0.01$, $^{***}P < 0.001$

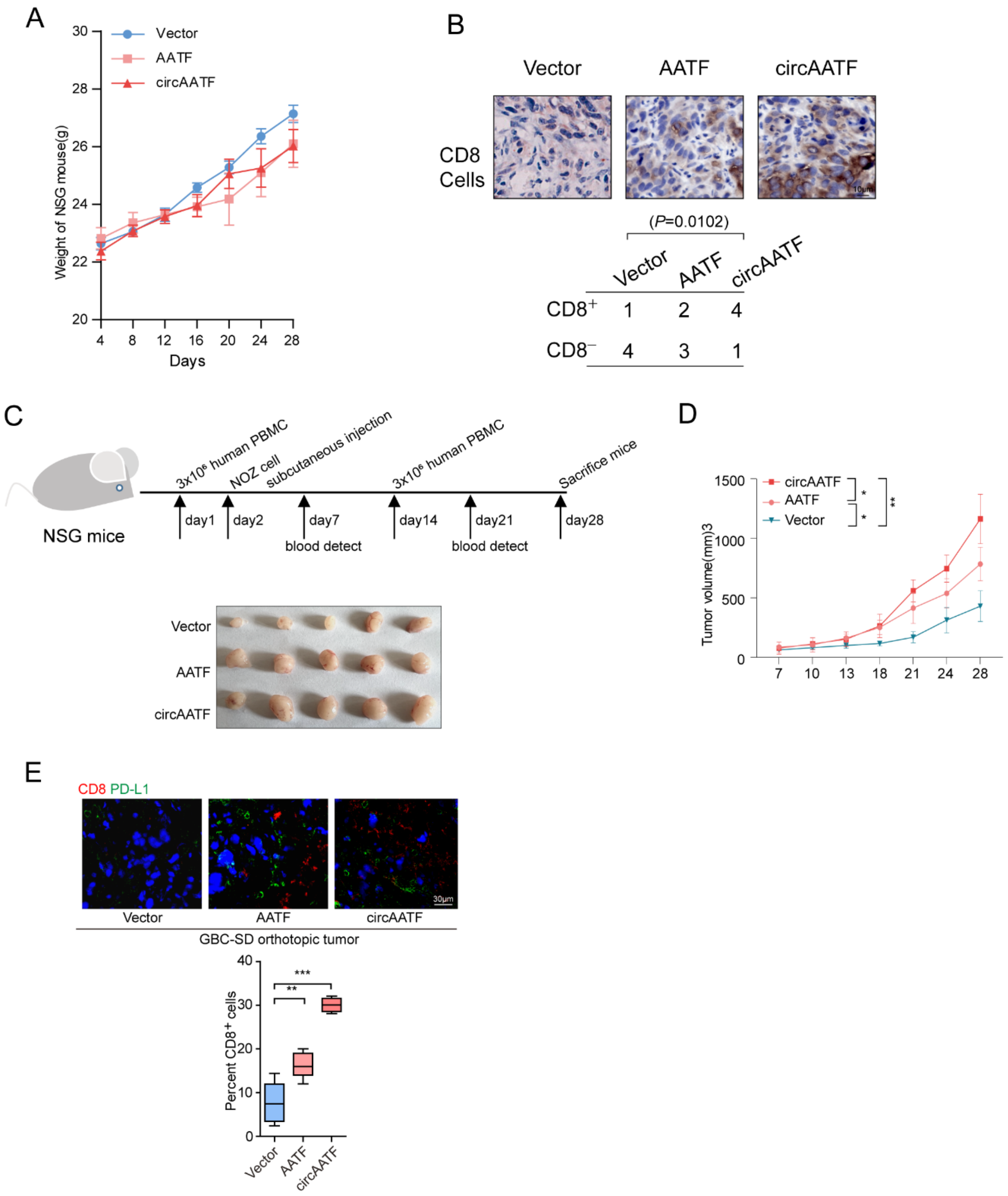

Supplementary Figure S6. The association between the expression levels of circRNAs and T cell abundances in GBC. (**A**) Weight changes of NSG mice with transfection of circAATF, *AATF* and vector at different time points**.** (**B**) Representative immunohistochemistry (IHC) images showing CD8^{+} T cell abundance levels in GBC tumors from different mice groups. (**C**) Comparison of tumor volumes among mice groups injected with NOZ cells transfected with vector, *AATF* mRNA, and circAATF. (**D**) Quantification of tumor sizes as shown in (**C**). (**E**) Fluorescence immunohistochemistry results showing CD8^{+} T cell and PD-L1^{+} cell abundance levels in cells transfected with circAATF, *AATF*, and vector. $^{*}P < 0.05$, $^{**}P < 0.01$, $^{***}P < 0.001$.

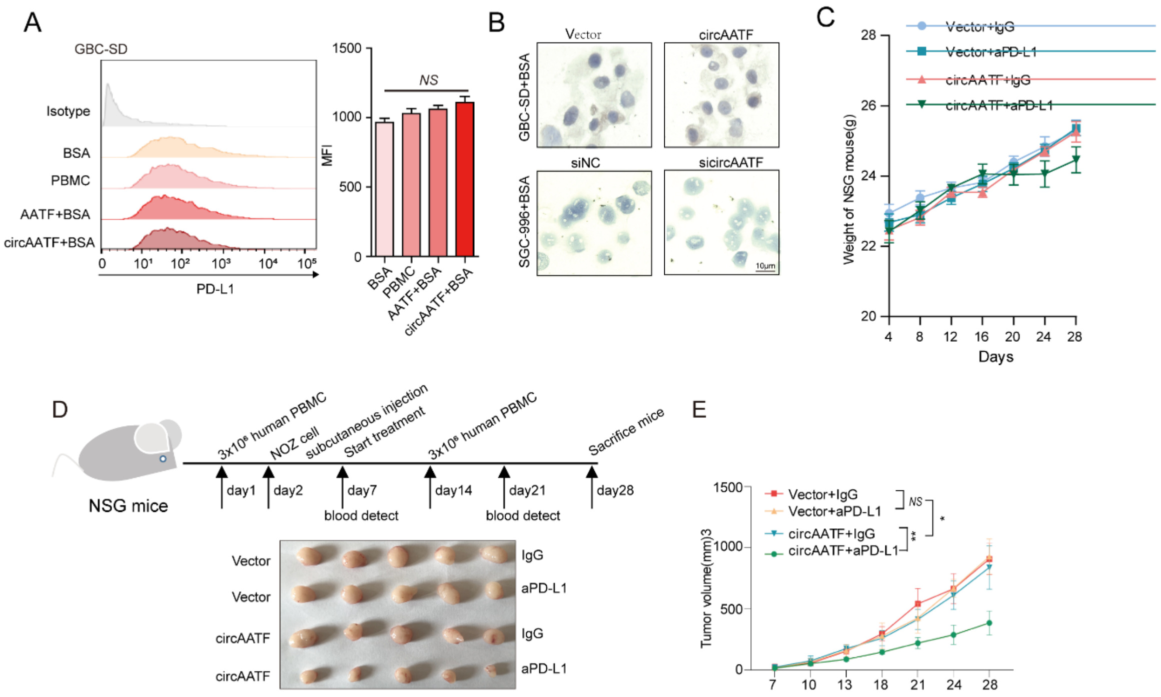

Supplementary Figure S7. Evaluation of PD-L1 expression under different conditions. (**A**) Flow cytometry analysis of PD-L1 expression in circAATF with BSA (circAATF+BSA), *AATF* with BSA (*AATF*+BSA), PBMC, and BSA alone. (**B**) IHC analysis of PD-L1 expression in GBC-SD cells with BSA and circAATF overexpression, and in SGC-996 cells with BSA and circAATF knockdown. (**C**) Weights of NSG mice as shown in Figure 6G. (**D**) Comparison of tumor responses among different anti-PD-L1 treated mice groups injected with the NOZ cells transfected with either vector or circAATF. (**E**) Quantification of tumor sizes as shown in (**D**). $^{*}P < 0.05$, $^{**}P < 0.01$

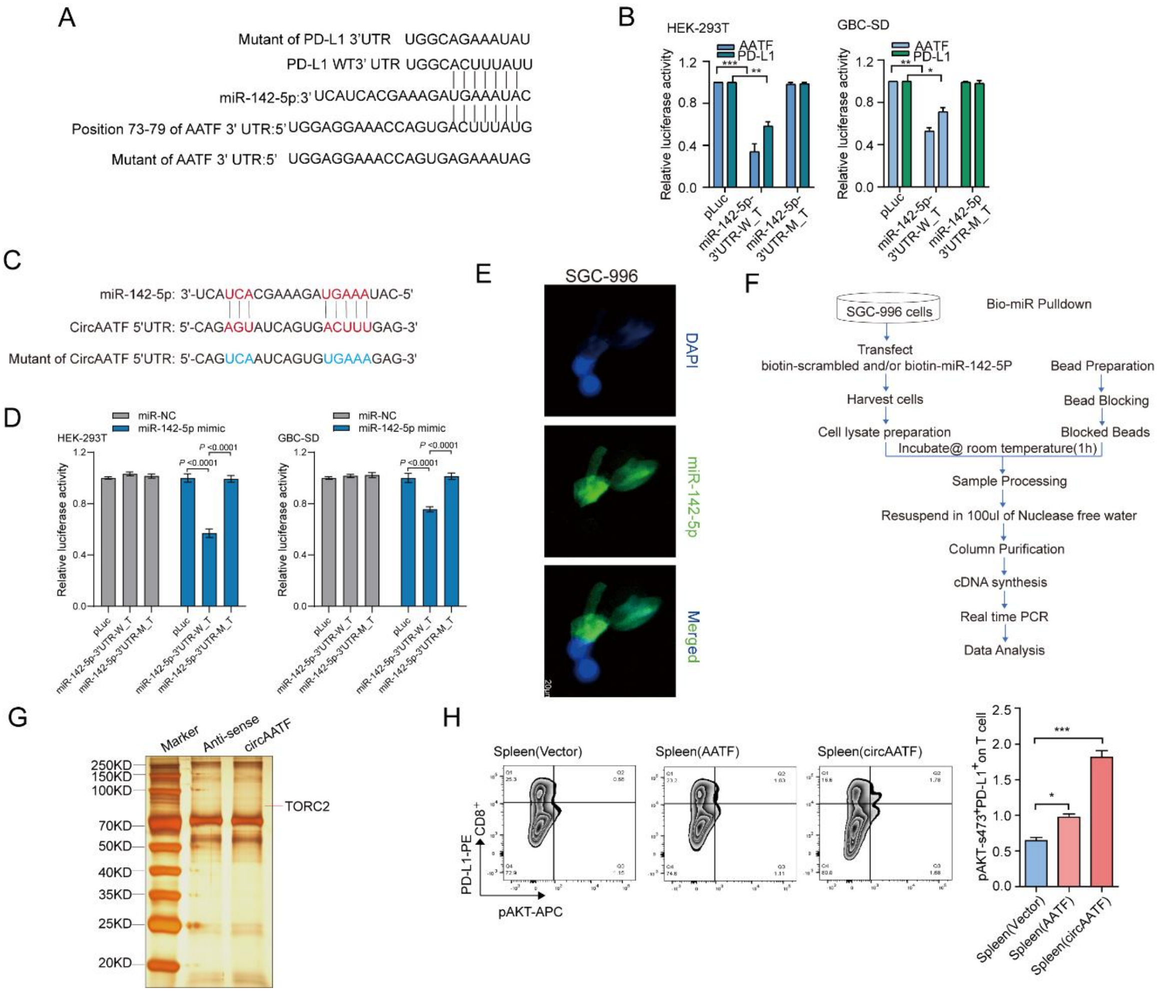

Supplementary Figure S8. CircAATF regulates PD-L1 through pAKT and *AATF*. (**A**) Target sequences shared by miR-142-5p in both *AATF* and PD-L1 mRNA. (**B**) Relative expression levels of *AATF* and PD-L1 in the HEK-393T and GBC-SD cell lines transfected with 3'UTR mutant and wild-type *AATF* and PD-L1, as well as controls. (**C**) The binding sites of miR-142-5p on circAATF. (**D**) Relative luciferase activity levels in HEK-293T and GBC-SD cell lines transfected with 3' UTR mutant and wild-type circAATF, miR-142-5p, and miR-NC. (**E**) FISH experiment validates the nuclear localization of miR-142-5p in the SGC-996 cell lines. (**F**) Schematic of the Bio-miR pulldown assay. (**G**) Pulldown results of circAATF. (**H**) Flow cytometry analysis of PD-L1 and pAKT expression levels in $CD8^+$ T cells, with the according quantification bar plot also shown. $^{*}P < 0.05$, $^{**}P < 0.01$, $^{***}P < 0.0001$

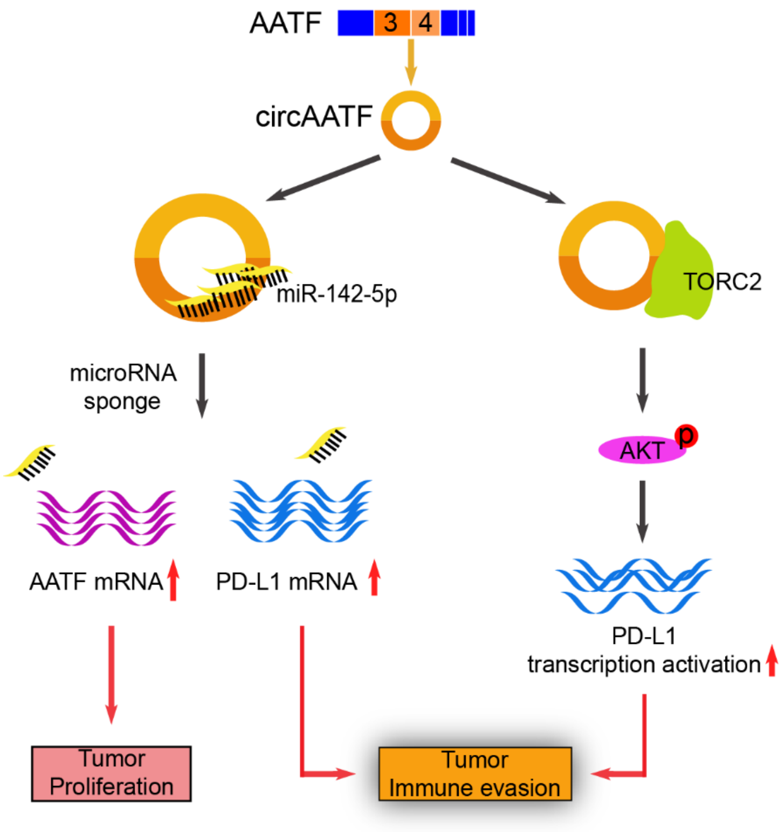

Supplementary Figure S9. Graphic summary of the circAATF-mediated regulatory network in GBC.

Supplementary Methods

RNA-seq data processing

Raw sequencing reads were initially processed to remove adapter sequences and low-quality bases using the Trimmomatic (Version 0.36) software[1] with such parameters as "*ILLUMINACLIP: 'Aapter':2:30:10' LEADING:3 TRAILING:3 SLIDINGWINDOW:4:15 MINLEN:36*". Subsequently, all filtered reads were aligned to the human reference genome (GRch38) using the splice-aware aligner HISAT2[2] with default settings. SAMtools (version 1.9)[3] was subsequently used to process the aligned reads, extracting those that could not be directly mapped to the reference genome. These unmapped reads were retained for subsequent back-splicing detection. Finally, the alignments were analyzed using the StringTie[4] program to calculate gene expression levels in TPM units (TPM = Transcripts per kilobase Per Million mapped reads). Genes annotated in GENCODE v28 were utilized to quantify gene expression.

GSEA analysis of GBC circRNAs

Protein-coding genes of interest were ranked based on the normalized back-splicing reads of circRNAs, which were derived from the corresponding genes. Subsequently, the ranked gene list was subjected to Gene Set Enrichment Analysis (GSEA)[5] to explore enriched hallmark gene sets.

Subcellular fractionation

The nuclear and cytoplasmic fractions of GBC-SD cells were prepared and collected following the user's manual of the Nuclear/Cytoplasmic Isolation Kit (Thermo Fisher Scientific, Carlsbad, California, USA). Additionally, U2 small nuclear RNA (snRNA) was used as an endogenous control, for the nuclear fraction, while β-actin was used as an endogenous control for the cytoplasmic fraction.

RNA FISH

Specific probes targeting the circAATF sequence were utilized for *in situ* hybridization. PCR fragments containing the T7 promoter were amplified using primers specific to the circAATF back-splicing region (see Supplementary **Table S4** for primer sequences). Digoxin or Biotin-labeled RNA probes were transcribed from PCR fragments using a Digoxin or Biotin RNA labeling mix and T7 RNA polymerase (Roche, Indianapolis, IN, USA). GBC-SD cells were cultured to the exponential phase and were 80-95% confluent

at the time of fixation. Following pre-hybridization with 1×PBS/0.5% Triton X-100, cells were hybridized in a hybridization buffer containing 40% formamide, 10% Dextran sulfate, 1×Denhardt's solution, 4×SSC, 10mM DDT, 1 mg/ml yeast tRNA, and 1 mg/ml sheared salmon sperm DNA, with Digoxin-labelled probes specific to circAATF, at 60 °C overnight. Signals were detected using a tyramide-conjugated Alexa 488 fluorochrome TSA kit (Life Technologies, Carlsbad, CA, USA). A double FISH assay was conducted on GBC-SD cells following co-transfection with circAATF. Biotin-labelled probes specific to circAATF (Exiqon, Vedbaek, Denmark) were used for hybridization. The signals from biotin-labeled probes were detected using Cy5-Streptavidin (Life Technologies, Carlsbad, CA, USA). Nuclei were counterstained with 4,6-diamidino-2-phenylindole (DAPI). Images were acquired using a Leica SP5 confocal microscope (Leica Micosystems, Mannheim, Germany). All experimental procedures were performed according to the manufacturer's instructions.

CCK-8 assays

Cells were seeded in 96-well flat-bottomed plates at a density of 1,500 cells per well in 100 μl of cell suspension. After a set period of culture, cell viability was measured using the Cell Counting Kit-8 (CCK-8) assay (Dojindo, Kumamoto Prefecture, Japan). Additionally, each experiment was performed in six replicates, repeated three times, and the measurements were taken continuously for 5 days.

Colony formation assays

Culture dishes of 6-well plates were used to seed 1,500 cells per well. The cells were cultured in a complete growth medium and allowed to grow until visible colonies formed, typically within10 to 14 days. Megascopic cell colonies were then fixed with methanol, stained with crystal violet (Sigma-Aldrich, St. Louis, MO), and counted.

Western blotting assays

Proteins were separated by sodium dodecyl sulfate-polyacrylamide gel electrophoresis (SDS-PAGE) and transferred onto nitrocellulose membranes (Bio-Rad, Hercules, CA, USA). The membranes were blocked with 5% nonfat milk and incubated with the corresponding primary antibodies, including AATF, phospho-AKT(S473), AKT, and PD-L1. This was followed by incubation with horseradish-peroxidase-conjugated secondary antibodies. The immunoreactivity was visualized using chemiluminescence and enhanced

chemiluminescence (ECL) reagents (Pierce Biotechnology, Rockford, IL, USA). Densitometry analysis was performed using Image-Pro Plus 6.0 software (Media Cybernetics, Rockville, MD, USA).

Quantitative Real-Time PCR

Total RNA samples from cell lines and clinical tissues were isolated using TRIzol reagent (Life Technologies, Carlsbad, CA, USA). For RNase R treatment, 2 mg of total RNA was incubated for 20 minutes at 37 °C with or without 3U/mg of RNase R (Epicentre Technologies, Madison, WI, USA), and the treated RNA samples were subsequently purified using the RNeasy MinElute Cleanup Kit (Qiagen). To quantify the levels of mRNA and circRNA, cDNA was synthesized from 500 ng of RNA samples using the PrimeScript RT Master Mix (Takara, Dalian, China). Real-time PCR analyses were conducted using SYBR Premix Ex Taq II (Takara). For circRNAs, divergent primers that anneal at the distal ends of circRNAs were employed to quantify their abundance. To determine the absolute amount of RNA, the purified PCR products amplified from cDNA corresponding to the circAATF sequence were serially diluted to generate a standard curve. The primers used for qRT-PCR are listed in Supplementary **Table S5**.

Estimation of immune cell abundance from gene expression profiles

For each sample, the CIBERSORT algorithm[6] was utilized to estimate the relative abundances of various immune cells from gene expression profiles. Specifically, CIBERSORT deconvolves immune-cell-type gene expression based on predefined gene signatures of different immune cell types. In our study, the LM22 gene signature was employed, which has been validated to exhibit significantly differential expression in one leukocyte population in contrast to all other haematopoietic cell populations. All T cell types within the LM22 signature were included in our analysis, encompassing "T cells CD4 memory activated", "T cells CD4 naive", "T cells CD4 memory resting", "T cells CD8", "T cells follicular helper", "T cells regulatory (Tregs)", and "T cells gamma delta".

Northern blotting assays

AATF RNA levels were determined using the Ambion Northern Max-Gly Kit (Thermo Fisher Scientific, Austin, TX, USA) in various tissues and cell lines. Initially, the extracted total RNA samples were electrophoresed and then transferred onto a positively charged nylon membrane. UV cross-linking was employed to secure the RNA onto the

nitrocellulose membrane. Subsequently, the AATF signal was detected using the DIG Northern Starter Kit (Roche, Indianapolis, IN, USA) with a digoxigenin-labeled AATF-specific oligonucleotide probe.

RNA interference and generation of lentiviral particles

The sequences of small interfering RNA (siRNA) oligonucleotides targeting circAATF, and the negative control siRNA, were purchased from RiboBio Co., Ltd (Guangzhou, China) and are listed in Supplementary **Table S3**. Transfections with siRNA (75 nM) were performed using Lipofectamine 2000 (Invitrogen). The human circAATF sequence was cloned from the SGC-996 cell cDNA and then cloned into the BamHI and EcoRI sites of the lentivirus expression vector pWPXL to generate pWPXL-circAATF and pWPXL-AATF. The SGC-996 cells were transfected with pWPXL-circAATF and pWPXL-AATF, along with the packaging and envelope plasmids psPAX2 and pMD2.G, respectively, using Lipofectamine 2000 (Invitrogen) following the manufacturer's instructions. The virus particles were collected 48 hours after the transfection. SGC-996 cells were infected with recombinant lentivirus transducing units using 1 μg/ml polybrene (Sigma-Aldrich).

Luciferase reporter assays

Approximately 5,000 HEK-293T cells or 10,000 SGC-996 cells per well were plated into 96-well plates and co-transfected with 50 nmol/L miR-142-5p mimic (or NC), 50 ng of the luciferase reporter, and 10 ng of the pRL-CMV Renilla luciferase control reporter using 0.5 μL Lipofectamine 2000 (Invitrogen, Carlsbad, CA, USA) per well. After a 48-hour transfection, luciferase activities were quantified using a dual-luciferase reporter assay system (Promega, Madison, WI, USA).

Functional enrichment analysis

The list of unique genes associated with differentially expressed circRNAs was extracted to determine the enrichment in biological hallmark gene sets. A hypergeometric test was conducted to calculate the enrichment significance. The probability *P* was computed to assess the enrichment significance, as detailed below[7]:

$$\mathrm{P} = 1 - \mathrm{F}(x|N,K,M)$$
$$= 1 - \sum_{t=0}^{x} \frac{\binom{K}{t}\binom{N-K}{M-t}}{\binom{N}{M}}$$

where N represents the total number of all protein-coding genes annotated in GENCODE (v28), K denotes the number of genes within the hallmark under investigation, M signifies the number of circRNA-associated genes under analysis, and x is the count of genes intersecting between the investigated hallmark and the circRNA-associated genes.

Immunohistology staining

Formalin-fixed, paraffin-embedded lung and liver tissue sections were stained with hematoxylin and eosin (H&E; Sigma Chemical, St Louis, MO). Frozen tumor sections were fixed with cold acetone, a mixture of acetone and chloroform (1:1), and acetone. Tissue sections were blocked with a blocking buffer containing 5% normal horse serum and 1% normal goat serum in phosphate-buffered saline solution (PBS) and incubated with a rat anti-human CD4 antibody (Catalog # MA5-12259; Thermo Fisher Scientific). For PD-L1 staining, sections were blocked with a buffer containing 0.1% Triton X-100 for 40 minutes and then incubated with a rat anti-CD274 antibody (ABIN185339; Antibodies-Online) overnight at 4 °C. On the following day, tissue sections were incubated with a goat anti-rat horseradish peroxidase-conjugated secondary antibody (Life Technologies) for 1 hour at room temperature, followed by DAB staining for 5 to 10 minutes at room temperature. Nuclei were then counterstained with hematoxylin (Sigma Chemical), and tumor sections were mounted using ClearMount Mounting Solution (Life Technologies). Slides were visualized under a Nikon eclipse Ti fluorescence microscope (Nikon Instruments, Melville, NY). The intensity of immunostaining was assessed by two independent pathologists blinded to the clinicopathological data. A semi-quantitative H-score, ranging from 0 to 300, was calculated by multiplying the staining intensities (0: negative, 1: weak, 2: moderate, 3: strong) by the percentage of positive staining cancer cells (0-100 %) at each intensity level for each sample.

Immunoprecipitation assays

The indicated plasmids were transfected into GBC-SD cells, which were then co-cultured with PBMCs. Cells were lysed in RIPA buffer (Beyotime Biotechnology) supplemented with protease inhibitors and RNase Inhibitor (Life Technologies) and centrifuged at 16,400×g for 15 minutes. Supernatants were incubated with anti-AKT antibody (Cell Signaling Technology, #4691) or IgG control antibody (Cell Signaling Technology, #3900) overnight at 4 °C with gentle rotation. The beads were then washed three times with NT2

buffer (50 mM Tris-HCl, pH 7.4, 150 mM NaCl, 0.05% Nonidet P-40, 1 mM MgCl2) containing protease inhibitors and RNase Inhibitor (Thermo Fisher Scientific Inc.), followed by two washes with PBS containing the same inhibitors. After washing, proteins were eluted by competition with AKT peptides. The immunocomplexes were then resolved by SDS/PAGE and immunoblotting with an anti-PD-L1 antibody.

References

1 Bolger, A. M., Lohse, M. & Usadel, B. Trimmomatic: a flexible trimmer for Illumina sequence data. *Bioinformatics* **30**, 2114-2120 (2014).

2 Kim, D., Langmead, B. & Salzberg, S. L. HISAT: a fast spliced aligner with low memory requirements. *Nat Methods* **12**, 357-360 (2015).

3 Li, H. *et al.* The Sequence Alignment/Map format and SAMtools. *Bioinformatics* **25**, 2078-2079 (2009).

4 Pertea, M. *et al.* StringTie enables improved reconstruction of a transcriptome from RNA-seq reads. *Nat Biotechnol* **33**, 290-295 (2015).

5 Subramanian, A. *et al.* Gene set enrichment analysis: a knowledge-based approach for interpreting genome-wide expression profiles. *Proc Natl Acad Sci U S A* **102**, 15545-15550 (2005).

6 Newman, A. M. *et al.* Robust enumeration of cell subsets from tissue expression profiles. *Nat Methods* **12**, 453-457 (2015).

7 Li, S., Hu, Z., Zhao, Y., Huang, S. & He, X. Transcriptome-Wide Analysis Reveals the Landscape of Aberrant Alternative Splicing Events in Liver Cancer. *Hepatology* **69**, 359-375 (2019).
